# Genome-Wide Screen Reveals Replication Pathway for Quasi-Palindrome Fragility Dependent on Homologous Recombination

**DOI:** 10.1371/journal.pgen.1003979

**Published:** 2013-12-05

**Authors:** Yu Zhang, Natalie Saini, Ziwei Sheng, Kirill S. Lobachev

**Affiliations:** School of Biology and Institute for Bioengineering and Bioscience, Georgia Institute of Technology, Atlanta, Georgia, United States of America; Duke University, United States of America

## Abstract

Inverted repeats capable of forming hairpin and cruciform structures present a threat to chromosomal integrity. They induce double strand breaks, which lead to gross chromosomal rearrangements, the hallmarks of cancers and hereditary diseases. Secondary structure formation at this motif has been proposed to be the driving force for the instability, albeit the mechanisms leading to the fragility are not well-understood. We carried out a genome-wide screen to uncover the genetic players that govern fragility of homologous and homeologous *Alu* quasi-palindromes in the yeast *Saccharomyces cerevisiae*. We found that depletion or lack of components of the DNA replication machinery, proteins involved in Fe-S cluster biogenesis, the replication-pausing checkpoint pathway, the telomere maintenance complex or the Sgs1-Top3-Rmi1 dissolvasome augment fragility at *Alu*-IRs. Rad51, a component of the homologous recombination pathway, was found to be required for replication arrest and breakage at the repeats specifically in replication-deficient strains. These data demonstrate that Rad51 is required for the formation of breakage-prone secondary structures in situations when replication is compromised while another mechanism operates in DSB formation in replication-proficient strains.

## Author Summary

Inverted repeats are found in many eukaryotic genomes including humans. They have a potential to cause chromosomal breakage and rearrangements that contribute to genome polymorphism and the development of diseases. Instability of inverted repeats is accounted for by their propensity to adopt DNA secondary structures that is negatively affected by the distance between the repeats and level of sequence divergence. However, the genetic factors that promote the abnormal structure formation or affect the ability of the repeats to break are largely unknown. Here, using a genome-wide screen we identified 38 mutants that destabilize imperfect human inverted *Alu* repeats and predispose them to breakage. The proteins that are required to maintain repeat stability belong to the core of the DNA replication machinery and to the accessory proteins that help replication fork to move through the difficult templates. Remarkably, when replication machinery is compromised, the proteins involved in homologous recombination promote the formation of secondary structures and replication block thereby triggering breakage at the inverted repeats. These results reveal a powerful pathway for the destabilization of chromosomes containing inverted repeats that requires the activity of homologous recombination.

## Introduction

Long palindromic sequences (inverted repeats ∼100 bp or more each without a spacer or with a short spacer) present a threat to both prokaryotic and eukaryotic genome stability. In *E. coli*, long palindromes placed on plasmids are frequently excised and cause cell inviability when introduced to chromosome [1]. In yeast, they have been shown to drastically induce ectopic and allelic recombination and a variety of gross chromosomal rearrangements (GCRs) including deletions, translocations and gene amplification [2]–[9]. Long inverted repeats were demonstrated to undergo frequent deletions and induce gene conversion and intra-chromosomal recombination in mice [10]–[12]. Palindromic sequences have been found in the vicinity of chromosomal breakpoints of translocations in humans and are implicated in the pathogenesis of diseases. For example, palindromic AT-rich repeats (PATRRs) have been shown to induce both non-recurrent and recurrent translocations; the latter could result into Emanuel syndrome [13]–[18]. Palindrome-mediated large deletions and interchromosomal insertions are causative factors of several types of εγδβ thalassemia [19] and X-linked congenital hypertrichosis syndrome, respectively [20]. Also, palindromes are abundant in cancer cells and are associated with DNA amplification in colon and breast cancer, medulloblastoma and lymphoma [21]–[28].

Palindromic sequences can form hairpin and cruciform structures due to their intrinsic symmetry [1]. Formation of these aberrant structures has been considered to be responsible for the genetic instability associated with this sequence motif. Hairpins occurring on the lagging strand can interfere with DNA replication and be attacked by structure-specific nucleases leading to DSBs. In *E. coli*, hairpins formed during DNA replication at long palindromic repeats are cleaved by the SbcDC nuclease [29]–[33]. Similarly, in *S. pombe*, the nuclease activity of the Mre11/Rad50/Nbs1complex (Mre11/Rad50 is the homolog of SbcDC) was implicated in the generation of breaks at palindromes [8], [34]. However, Casper et al. (2009) showed that in *S. cerevisiae*, the Mre11 complex is not involved in breakage at a large inverted repeat consisting of two Ty1 elements with a ∼280 bp spacer in strains where DNA polymerase α was down-regulated [35]. We previously demonstrated that in *S. cerevisiae*, the Mre11/Rad50/Xrs2 complex does not initiate DSBs at closely spaced *Alu* inverted repeats (*Alu*-IRs) but is required along with Sae2 for processing breaks that have hairpin termini [5]. This disparity in the Mre11 complex's effect on DSB generation at palindromic sequences might be attributed to the difference in the formation of stable hairpins during replication and the inability of this complex to cleave hairpins with large loops. This conjecture, however, remains to be experimentally proven. These observations also point out the existence of an Mre11-independent pathway in generating DSBs at palindromic sequences. We proposed that in yeast, *Alu*-IR-mediated hairpin-capped breaks can result from the resolution of cruciform structures in which a putative nuclease cleaves symmetrically at the base of the two hairpins [5]. Cruciform resolution on plasmid in yeast was shown to be dependent on the structure-specific endonuclease Mus81/Mms4 [36], although chromosomal fragility at inverted repeats was not influenced by this complex [5]. Cruciform formation and resolution were also proposed to be the triggering events for translocations at PATRRs in human sperm cells [37]–[39]. Recently, in a plasmid transfection assay, the GEN1 nuclease was implicated in cruciform resolution in HEK293 cells, and the resultant hairpin-capped breaks were further processed by Artemis for DSB repair [40]. Whether this mechanism operates in PATRR-mediated chromosomal translocations remains to be established.

Although the formation of hairpin and cruciform structures is deemed as the key initiation event for fragility at inverted repeats, the pathways that predispose eukaryotic cells to or provide protection against chromosomal breaks are still not well defined. Previously, deficiencies in Pol1, Pol3 and Rad27 proteins responsible for synthesis of the lagging strand during DNA replication were found to augment instability at inverted repeats [3], [7], [9]. However, it is unknown if fragility is exclusively confined to deficiencies in lagging strand synthesis. In addition, it is important to identify mechanisms that facilitate or prevent instability of imperfect IRs that contain a spacer (quasi-palindrome) and are not fully homologous to each other, since these repeats prevail over perfect palindromes in the human genome [9], [41].

In this study, we carried out an unbiased genome-wide screen aimed at identifying the genetic factors controlling fragility of homologous and divergent *Alu*-quasi-palindromes in yeast. Using 12 bp-spaced *Alu*-IRs with either 100% or 94% homology between the two repeats, we analyzed the effects of deletions of around 4800 non-essential genes and downregulation of 800 essential genes on quasi-palindrome-mediated GCRs. In addition to defects in lagging strand synthesis, we found that deficiencies in proteins involved in replication initiation and leading strand synthesis, replication pausing checkpoint pathway, the Sgs1-Top3-Rmi1 dissolvasome, proteins involved in Fe-S cluster biogenesis or telomere maintenance augment breakage and GCRs induced by *Alu*-IRs. Replication block and fragility at inverted repeats in replication-deficient strains were abrogated upon deletion of *RAD51*, indicating an unexpected role for homologous recombination in the formation of cruciform structure at palindromic repeats when replication is compromised.

## Results

### Experimental systems used in the genome-wide screen

**Figure 1:**
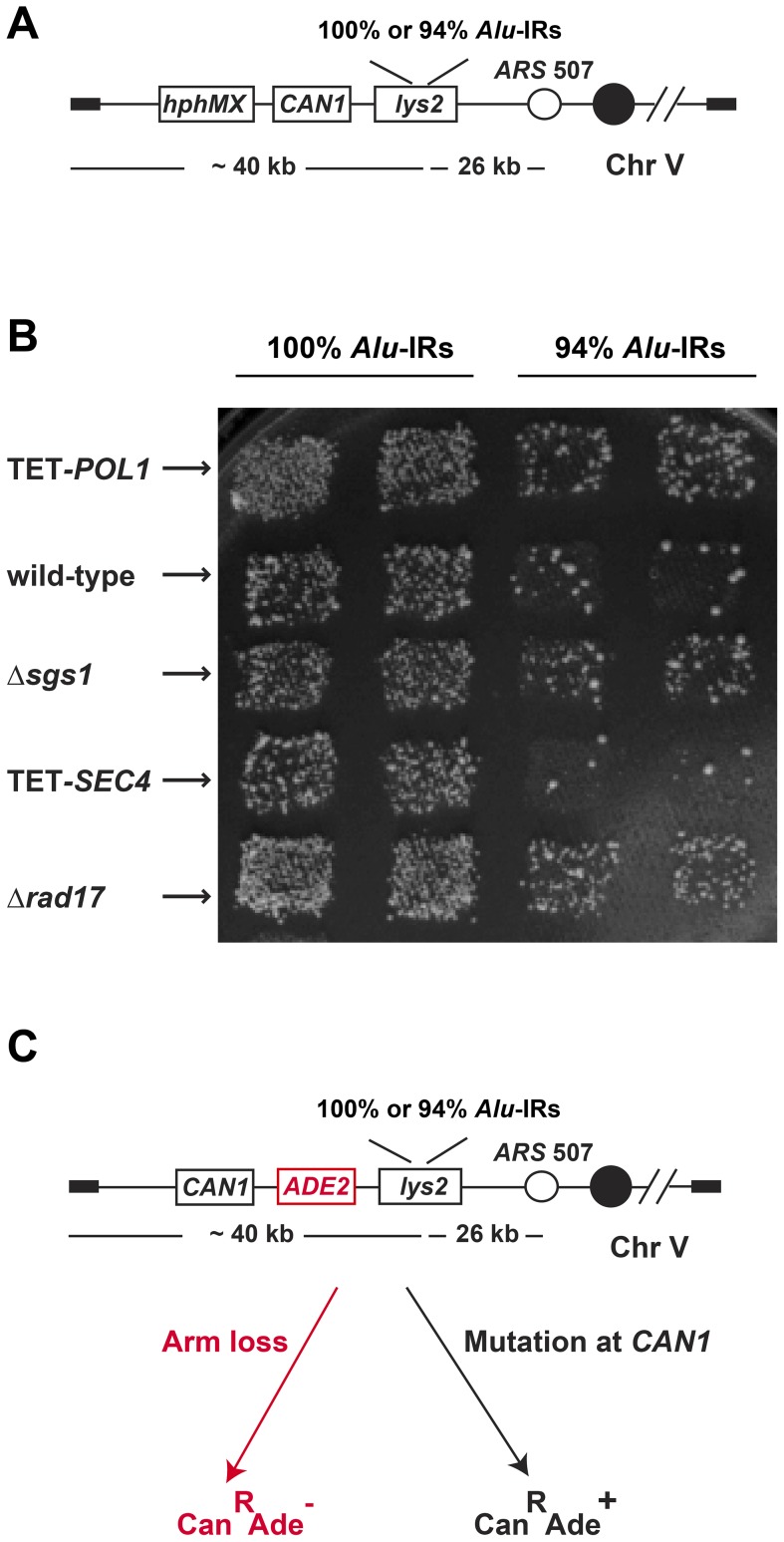
The genome-wide screen to identify hyper-GCR mutants. (**A**) Experimental construct in the query strains. 100% homologous or 94% homeologous *Alu*-IRs were inserted into the left arm of chromosome V. The selectable marker *hphMX* for the presence of the chromosomal fragment containing the repeats and the counterselectable marker *CAN1* used to assay GCR events are depicted. (**B**) Representative plate showing papillae on canavanine-containing medium reflecting the levels of *Alu*-IR-induced GCRs in wild-type and mutants. Columns are duplicates of query strains containing 100% homologous or 94% homeologous *Alu*-IRs. Each row is a tester strain containing the corresponding mutation. (**C**) Experimental construct for verifying the effect of hyper-GCR mutants obtained from the screen. *ADE2* was inserted between *CAN1* and the repeats. As a result, GCR events appear as red Can^R^ colonies and mutations at *CAN1* give rise to white Can^R^ colonies on canavanine-containing medium with low amounts of adenine.

We systematically analyzed the effect of more than 6000 mutations on *Alu*-IR-mediated fragility using a genome-wide screen in the yeast *S. cerevisiae* (Figure 1 and Figure S1). The screen's scheme is based on the approach developed in Tong et al., 2001 [42] with modifications. In the query strains, a quasi-palindrome consisting of two 320 bp *Alu* elements in inverted orientation with a 12 bp spacer was placed telomere-distal to the counterselectable marker *CAN1* on the left arm of chromosome V. The two *Alu* elements were either 100% or 94% homologous (100% *Alu*-IRs or 94% *Alu*-IRs). Breakage at the *Alu*-IRs and loss of the 40 kb telomere-proximal fragment results in canavanine-resistant colonies. The tester strains included a complete set of 4786 deletion mutations for non-essential genes (YKO strains) and two sets of 842 essential genes whose expression is either regulated by the doxycycline-repressible promoter (yTHC strains) or decreased due to mRNA perturbation (DAmP strains). An *hphMX* cassette was positioned telomere-proximal to the *Alu*-IRs, providing a marker for selecting the presence of the repeats during the screening and the testers were marked by a *kanMX* cassette. The schematics for combining the left arm of chromosome V containing the fragile motifs and the mutations have been previously applied to study instability of the trinucleotide GAA/TTC repeats and are described in detail in Zhang et al., 2012 [43]. Briefly, the query strains were crossed with each tester strain to get diploids, which then underwent sporulation. Haploids containing both the *Alu*-IRs and the mutation of interest were replica plated to canavanine-containing medium. Mutants with augmented repeat-induced GCRs exhibited increased number of canavanine-resistant papillae compared to the wild-type strains. Since the rate of canavanine-resistant colonies occurring due to GCR in the wild-type strain carrying 100% *Alu*-IRs is 10-fold higher (5×10^−5^) than in the strongest mutator Δ*msh2* (6×10^−6^), the screen specifically identified hyper-fragility mutants.

We verified the effect of the identified mutants by recreating the hyper-fragile alleles in strains with the *ADE2* gene inserted between *CAN1* and *Alu*-IRs that allows differentiation of GCRs from mutations based on the color of canavanine-resistant clones [6] (Figure 1). To create the mutant alleles, the *kanMX* cassette was used to knockout non-essential genes and a *tetO7* repressible promoter was used to replace the natural promoters of essential genes and regulate their expression [44]. The essential genes under the control of *tetO7* promoter will be referred to as TET-*ORF*s in the following text.

### Mutants with increased fragility at *Alu*-IRs

38 mutants that exhibit a hyper-fragility phenotype in strains containing either 100% or 94% homologous *Alu*-IRs were identified from the screen (Table S1). 17 mutants belonged to the YKO collection, 17 mutants were uncovered from the yTHC collection and 4 mutants were identified from the DAmP collection. The mutants could be grouped into six classes of genes coding for the dissolvasome and proteins involved in replication, Fe-S cluster biogenesis, checkpoint response, telomere maintenance and DSB repair.

Previously, it has been shown that downregulation of or mutation in the DNA polymerases α and δ causes increased instability of inverted repeats [3], [5], [7]. Consistently, we found that TET-*POL1* and TET-*POL3* strains destabilize both 100% and 94% *Alu*-IRs and exhibit 11- to 20-fold higher fragility than the wild-type strains. This screen also revealed that downregulation or deletion of other key components of the DNA replication pathway, namely, the origin recognition complex ORC, the DNA helicase Mcm2-7, the DNA primase complex, the leading strand synthesis polymerase ε, the single-strand binding protein RPA, the polymerase sliding clamp PCNA, the clamp loader RFCs or the endonucleases Dna2 and Rad27 participating in Okazaki fragment maturation, induce fragility at *Alu*-IRs. Deficiencies in these proteins caused a 3- to 15-fold and a 3- to 34-fold increase in GCR rates for 100% *Alu*-IRs and 94% *Alu*-IRs, respectively. We also observed a 5- to 9-fold elevation of GCRs in strains carrying the defective replication checkpoint surveillance complex, Mrc1-Tof1-Csm3. This result prompted us to test if Mec1, which is recruited to stalled replication forks and phosphorylates Mrc1 in response to DNA replication stress [45], [46], senses inverted repeat-mediated replication impediment. Since Δ*mec1* is lethal, we assessed the effect of Δ*mec1* in Δ*sml1* background. We found that Δ*mec1*Δ*sml1* but not Δ*sml1* led to a 5-fold increase in GCRs. These data demonstrate that intact replication machinery and replication checkpoint are required to prevent palindrome instability. Moreover, secondary structure formation and breakage are not only restricted to defects in lagging strand synthesis since fragility is also increased in strains where Polε and Mcm2-7 complex were downregulated.

Besides the replication checkpoint surveillance mutants, the screen also revealed that GCRs mildly increase (2- to 4-fold) in Δ*rad17*, Δ*mec3*, Δ*ddc1* and Δ*rad24* mutants deficient in DNA damage checkpoint signaling [47]. As discussed below, this effect could be explained by the improved recovery of the broken chromosome when checkpoint activation is impaired.

The third group of mutants that amplify *Alu*-IRs fragility included members of the cytosolic iron-sulfur protein assembly targeting complex. TET-*YHR122W* led to a 3- and 8-fold increase in GCRs in 100% and 94% *Alu*-IRs, respectively. Yhr122w was shown to physically interact with Cia1 and Mms19 in the biogenesis of Fe-S clusters in various DNA repair and replication proteins [48], [49]. We found that disruption of *MMS19* led to an 18- and 14-fold increase in GCRs in strains containing 100% and 94% *Alu*-IRs, respectively. This is also consistent with our previous finding that Δ*mms19* causes an increase in *Alu*-IR-induced homologous recombination [9].

The screen revealed that deletion of *SGS1*, the RecQ helicase homolog implicated in the dissolution of branched DNA structures and unwinding of CTG/CAG hairpins [50], [51], caused a 10- and 7-fold elevation in GCRs in 100% and 94% repeats-containing strains. Sgs1 interacts with Rmi1 and Top3 to form the dissolvasome complex [52]. Consistently, we found that deletion of *RMI1* and of *YLR235C* that partially overlaps with *TOP3* also led to hyper-fragility (Table 1 and Table S1). Our data suggest potential roles of Sgs1-Rmi1-Top3 in influencing palindrome stability through unwinding the hairpin or cruciform structures formed by the repeats.

**Table 1:** Mutants with increased *Alu*-IR-induced GCR rate.

Genetic background	GCR rate (×10^−6^)			
	100% homologous		94% homologous	
wild-type	41 (30–52)^a^		5 (4–6)	
Replication mutants				
TET-*RFA2*	250 (100–280)	6^b^	170 (80–180)	34
TET-*POL2*	240 (210–270)	6	130 (90–150)	26
TET-*POL1*	470 (380–500)	11	100 (80–110)	20
TET-*POL3*	460 (390–640)	11	82 (72–102)	16
TET-*POL30*	370 (290–390)	9	69 (60–73)	14
TET-*RFC2*	280 (170–380)	7	34 (21–44)	6
TET-*YHR122W*	140 (110–160)	3	38 (23–47)	8
Δ*mms19*	720 (370–820)	18	72 (61–85)	14
TET-*PRI2*	340 (260–470)	8	170 (130–200)	34
TET-*MCM2*	150 (140–240)	4	41 (16–62)	8
TET-*ORC4*	110 (80–230)	3	62 (31–76)	12
TET-*DNA2*	120 (80–200)	3	9 (9–12)	3
Δ*rad27*	600 (450–920)	15	90 (60–240)	18
Δ*pol32*	240 (190–300)	6	32 (28–36)	6
Checkpoint response genes				
Δ*tof1*	250 (170–300)	6	39 (31–48)	8
Δ*csm3*	370 (270–530)	9	27 (22–37)	5
Δ*rad17*	180 (160–250)	4	14 (13–16)	3
Δ*rad24*	140 (130–190)	3	12 (11–17)	2
Δ*mec1*Δ*sml1*	200 (160–220)	5	ND^c^	ND
Δ*sml1*	43 (40–49)	1	ND	ND
Helicase				
Δ*sgs1*	410 (300–490)	10	35 (26–44)	7
Δ*rmi1*	480 (410–560)	12	ND	ND
Telomere protection genes				
TET-*TEN1*	140 (120–230)	3	13 (9–16)	3
TET-*CDC13*	120 (70–140)	3	14 (11–18)	3
Double strand breaks repair genes				
Δ*mre11*	420 (370–440)	10	210 (170–230)	42
Δ*rad50*	400 (370–430)	10	220 (200–250)	44

a Numbers in the brackets are 95% confidence intervals.

b Fold increase in GCR rates in mutants compared to wild-type strains.

c Not determined.

The fifth group of hyper-fragile mutants consisted of TET-*TEN1*, TET-*STN1* and TET-*CDC13*. The Ten1-Stn1-Cdc13 complex is involved in telomere maintenance and protection [53]. Downregulation of Ten1 resulted in a 3-fold elevation of fragility (Table 1). The TET-*CDC13* strain demonstrated a similar increase in the level of arm loss. Notably, the closest telomere is about 40 kb away from the location of the inverted repeats. In another study, we found that downregulation of Ten1-Stn1-Cdc13 also predisposes the triplex-forming GAA/TTC repeats to breakage and expansions [43]. Taken together, these data suggest among other possibilities that this complex plays a role in helping replication machinery to move through difficult regions.

Previously, we demonstrated that the Mre11-Rad50-Xrs2 complex and the Sae2 protein are required to open hairpins to initiate DSB repair at inverted repeats [5]. We also showed that in Δ*mre11* mutants, GCR rates increased likely due to the inability of mutants to hold DSB ends together and open the hairpin termini, which therefore increase the probability of formation of dicentric chromosomes [6]. Predictably, the screen identified Δ*mre11* and Δ*rad50* as hyper-fragile mutants with a 10- and ∼44-fold increase in GCRs induced by homologous and homeologous *Alu*-IRs, correspondingly. This group therefore encompasses mutants that do not impact secondary structure formation and breakage, but rather increase probability of arm loss and recovery of the broken chromosome.

### DSB formation is increased in replication-deficient and Δ*sgs1* mutants

In the wild-type strain, DSBs induced by *Alu*-IRs have covalently-closed hairpin termini [5]. To determine if the nature of breaks in the identified hyper-GCR mutants was similar to the wild-type strain, we characterized DSB intermediates in a subset of mutants. In addition, estimation of the level of breaks provides a way to distinguish between mutants that facilitate formation or enhance stability of the secondary structures and mutants that increase the loss of the acentric DSB fragment (e.g. *mrx* mutants) or improve the recovery of the broken chromosome.

**Figure 2:**
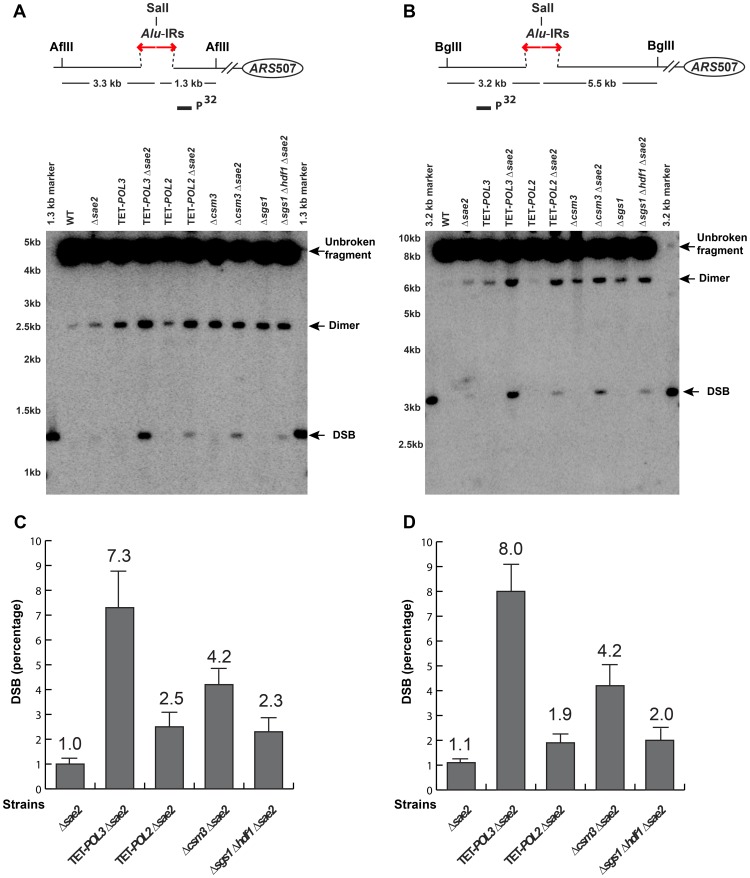
Physical detection of breakage intermediates in the wild-type and mutant strains carrying 100% *Alu*-IRs. Yeast genomic DNA embedded in agarose plugs was digested with either AflII (**A**) or BglII (**B**). The relative positions of the repeats, the restriction sites and the replication origin *ARS*507 are illustrated. Digested DNA was separated by gel electrophoresis. Southern hybridization using *LYS2*-specific probes (solid rectangles) was carried out to detect the chromosomal fragments centromere-proximal (**A**) or distal (**B**) to the breaks induced by *Alu*-IRs. For the centromere-proximal intermediates (**A**), the size of unbroken fragment, dimer and DSB fragment are 4.6 kb, 2.6 kb and 1.3 kb, respectively. For the centromere-distal intermediates (**B**), the size of unbroken fragment, dimer and DSB fragment are 8.7 kb, 6.4 kb and 3.2 kb, respectively. Bands corresponding to the unbroken fragment, dimer and DSB fragment are indicated by arrows. The 1.3 kb marker and 3.2 kb marker were generated by digesting genomic DNA from the wild-type *Alu*-IRs strain with SalI+AflII (**A**) or SalI+BglII (**B**), where SalI cuts inside the 12 bp spacer of the *Alu*-IRs. The strains used for analysis are: wild-type, Δ*sae2*, TET-*POL3*, TET-*POL3*Δ*sae2*, TET-*POL2*, TET-*POL2*Δ*sae2*, Δ*csm3*, Δ*csm3*Δ*sae2*, Δ*sgs1*, Δ*sgs1*Δ*hdf1*Δ*sae2*. (**C**) and (**D**) Densitometry analysis of the broken fragments normalized to the intact chromosome V in Δ*sae2* strains in (**A**) and (**B**), respectively. Values are shown as mean (shown on the top of the bars) with standard deviation obtained from at least three independent experiments.

We compared the levels of chromosomal breaks in the wild-type strain containing 100% *Alu*-IRs with a subset of mutants from each group described in the previous section (Figure 2 and Figure S2). DSB detection was carried out in Δ*sae2* strains to prevent the opening of the hairpins and the resection of the broken fragments [5]. The lethality of Δ*sgs1*Δ*sae2* can be rescued by the deletion of *HDF1* [54]. Therefore, the effect of Δ*sgs1* on DSB formation was assessed in the Δ*sgs1*Δ*sae2*Δ*hdf1* triple mutant. DSBs were analyzed with a telomere-distal probe upon AflII digestion or a telomere-proximal probe using BglII digestion of chromosomal DNA embedded in agarose plugs. Upon AflII or BglII digestion, DSBs occurring inside the repeats were expected to be 1.3 kb or 3.3 kb, respectively. We also anticipated the appearance of inverted dimers that are double the size of the DSB intermediates (2.6 kb or 6.6 kb, correspondingly). These molecules resulting from replication of hairpin-capped breaks were previously detected in the wild-type strains [5].

No DSBs were observed in the presence of Sae2 in both wild-type and mutant strains, likely due to hairpin opening and robust resection of the breaks. However, DSBs were readily detected in Δ*sae2* background. In TET-*POL3*, TET-*POL2*, Δ*csm3*, Δ*sgs1*Δ*hdf1* (Figure 2), Δ*mms19*, TET-*TEN1* (Figure S2) and Δ*sml1*Δ*mec1* (Figure S4) mutants, there was a 2- to 15- fold increase in breaks in comparison with wild-type strains when the telomere-proximal or the telomere-distal fragments were probed, indicating that these mutations increase fragility at *Alu*-IRs by either facilitating secondary structure formation or stabilizing the structures. It is important to note that no increase in DSBs were detected in the Δ*sae2*Δ*hdf1* and Δ*sae2*Δ*sml1* mutants (Figure S3 and Figure S4) indicating that the increase in fragility is due to deficiencies in Sgs1 and Mec1, accordingly. In Δ*rad17*, the amount of breaks was comparable to the wild-type strain, suggesting that DNA damage checkpoint-deficient mutants provide conditions for better recovery of the broken chromosomes, rather than affecting the formation and/or stability of the secondary structures. It is important to note that besides DSBs we could also detect dimers and no other intermediates were observed. The dependence of DSB detection on Δ*sae2* and the existence of dimers suggest that breaks in hyper-fragile mutants might contain hairpin termini similar to those in wild-type strains.

### DSBs in replication-deficient strains have hairpin-capped termini

**Figure 3:**
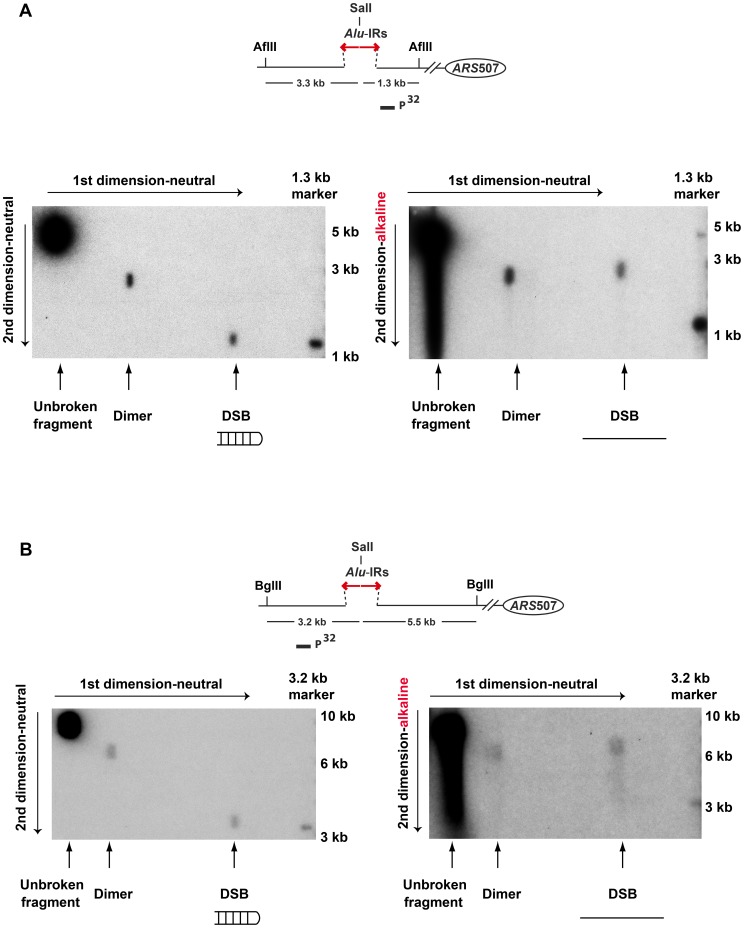
2D neutral/alkaline gel analysis of *Alu*-IR-induced DSBs in theTET-*POL3*Δ*sae2* strain. Yeast DNA embedded in agarose plugs was digested with AflII (**A**) or BglII (**B**). Digested DNA was separated in neutral conditions in the first dimension. In the second dimension, DNA was run in either neutral (left gel) or alkaline (right gel) conditions. Southern hybridization was done as described in Figure 2.

To test the premise of hairpin-capped breaks in the mutants experimentally, the DSB fragments in TET-*POL3*Δ*sae2* were analyzed via neutral/alkaline two-dimensional (2D) gel electrophoresis (Figure 3). We found that the 1.3 kb telomere-distal DSB fragment migrated as a 2.6 kb single-stranded DNA (ssDNA) fragment in the alkaline gel. Similarly, the 3.3 kb telomere-proximal DSB fragment migrated as a 6.6 kb ssDNA fragment under denaturing conditions. No additional bands (e.g. those corresponding to nicked hairpins) were seen, indicating that *Alu*-IRs generate covalently-closed hairpin-capped breaks in both TET-*POL3* and wild-type strains.

The symmetry of the breaks and the presence of covalently-closed hairpins at the DSB termini suggest that the final steps in breakage in mutants and wild-type are the same and include cruciform formation and resolution.

### *Alu*-IR-mediated fragility and fork arrest are Rad51-dependent in replication-deficient strains

**Figure 4:**
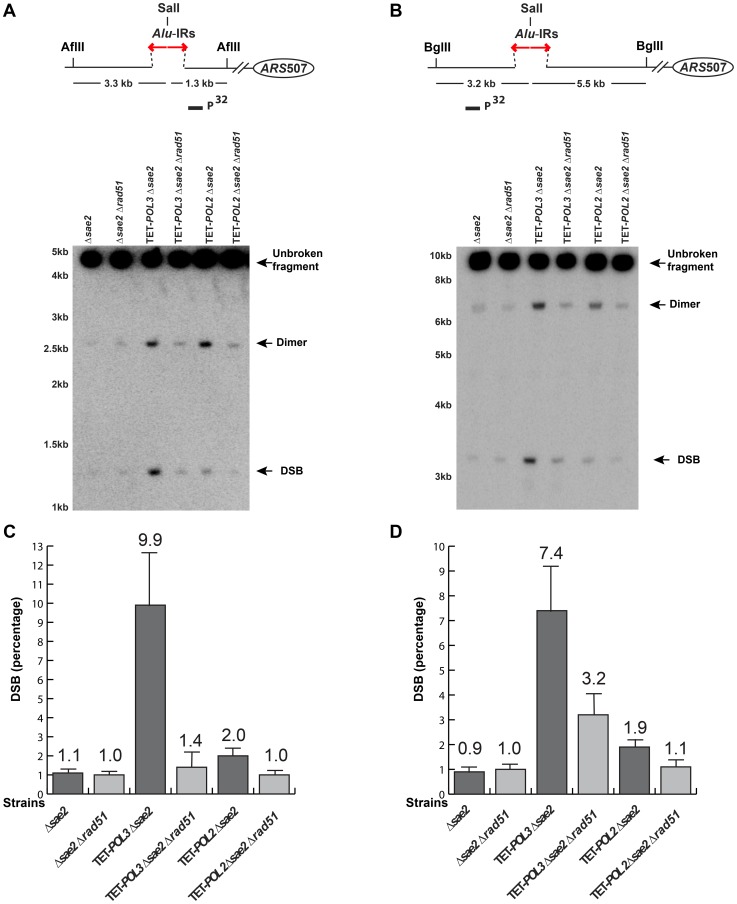
Detection of DSB accumulation in wild-type and mutant strains upon deletion of *RAD51*. DSB detection was carried out as described in Figure 2. The strains used in this analysis are: Δ*sae2*, Δ*sae2*Δ*rad51*, TET-*POL3*Δ*sae2*, TET-*POL3*Δ*sae2*Δ*rad51*, TET-*POL2*Δ*sae2*, TET-*POL2*Δ*sae2*Δ*rad51*. (**C**) and (**D**) Densitometry analysis of the broken fragments normalized to the intact chromosome V in Δ*sae2* strains in (**A**) and (**B**), respectively. Values are shown as mean (shown on the top of the bars) with standard deviation obtained from at least three independent experiments.

The screen revealed that mutants deficient in the DNA replication pathway comprise the major group that augments fragility at *Alu*-IRs. Analysis of DSB intermediates indicated that cruciform resolution is the likely scenario for fragility in these mutants (Figure 2, 3). Generation of ssDNA due to replication defects in the leading or lagging strands might provide optimal conditions for the formation of hairpins, not cruciforms. We hypothesized that a deficiency in the DNA replication can lead to formation of the cruciform structure through template switching when the fork stalls at a hairpin. In another screen for factors that channel replication stress into fragility, we identified Rad51, a key protein in homologous recombination. In the Δ*rad51* background, the GCR rates of both TET-*POL3* and TET-*POL2* mutants decreased almost to the wild-type level (Table 2). Consistent with the reduction in GCRs, the amount of DSBs and inverted dimers in TET-*POL3*Δ*sae2* and TET-*POL2*Δ*sae2* significantly decreased upon deletion of *RAD51*. Notably, lack of Rad51 does not affect GCR rates or DSB formation in the wild-type strains (Table 2 and Figure 4). Consistently, deletion of *RAD54*, the auxiliary protein for strand invasion during recombination, in wild-type and TET-*POL3* mutant had a similar effect on fragility (Table S2 and Figure S5) indicating that the involvement of homologous recombination in the induction of fragility is specific to conditions when replication is compromised.

**Table 2:** *Alu*-IR-mediated fragility in TET-*POL3* and TET-*POL2* mutants is Rad51-dependent.

Genetic background	GCR rate (×10^−6^)	Fold increase over wild-type
WT	41 (30–52)^a^	1
Δ*rad51*	37 (27–50)	1
TET-*POL3*	460 (390–640)	11
TET-*POL3*Δ*rad51*	88 (58–108)	2
TET-*POL2*	240 (210–270)	6
TET-*POL2*Δ*rad51*	63 (46–79)	1

a Numbers in brackets are the 95% confidence interval.

**Figure 5:**
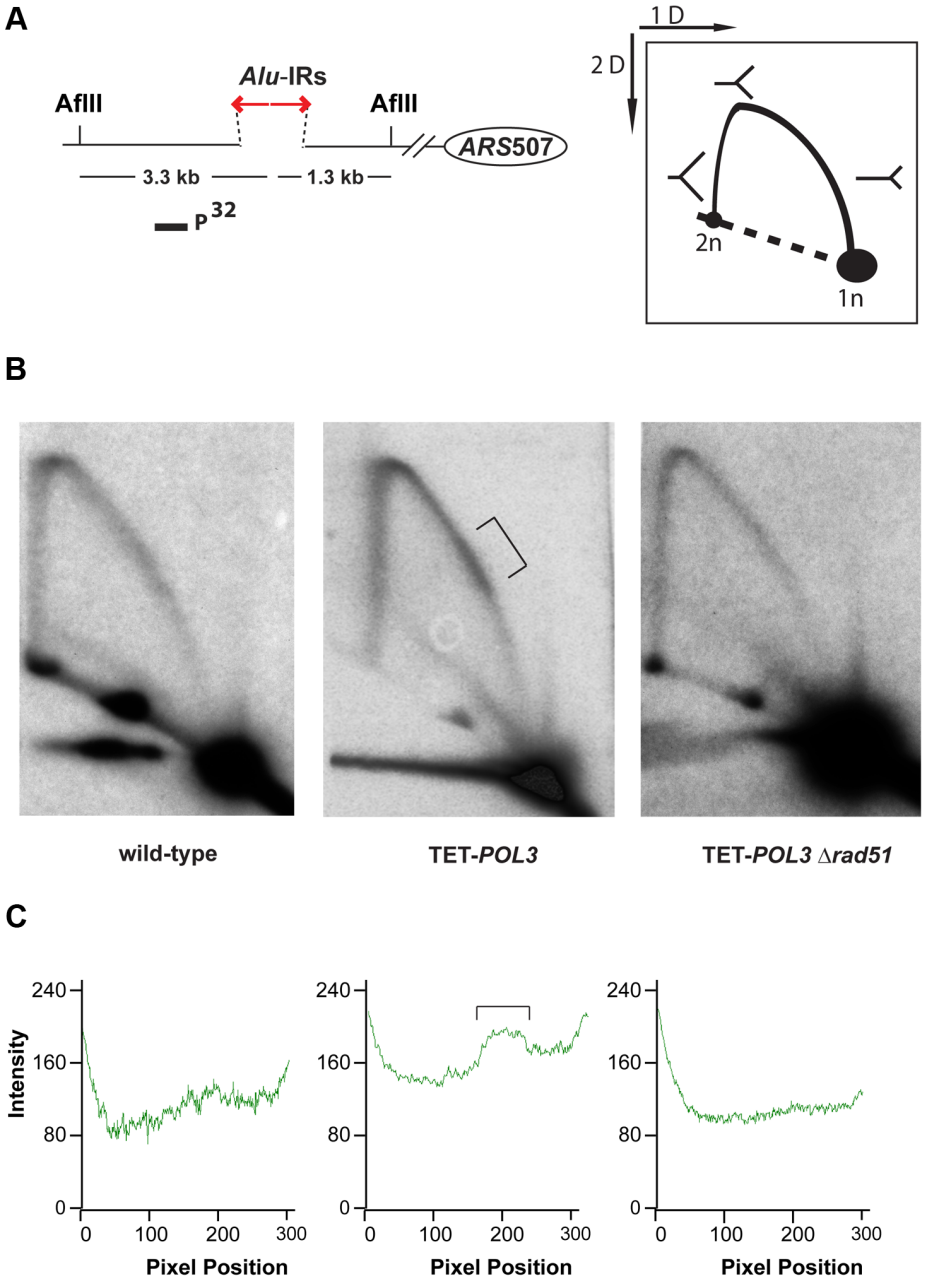
Analysis of replication fork progression through *Alu*-IRs in the wild-type and mutant strains. DNA samples from the wild-type, TET-*POL3* or TET-*POL3*Δ*rad51* strains were digested with AflII and processed for 2D gel analysis. (A) Illustration of restriction digestion and 2D gel analysis. The solid rectangle indicates the position of the probe used for Southern hybridization. (B) 2D gel analysis of replication intermediates in the wild-type and mutant strains. The zone of replication arrest in the TET-*POL3* strain is indicated by the bracket. (C) Densitometry analysis of the Y arc's long arm in the corresponding strains in (B). The relative radioactive counts along the long arm of the Y-arc that starts from the monomer are shown. The bracket depicts the zone of replication arrest.

**Figure 6:**
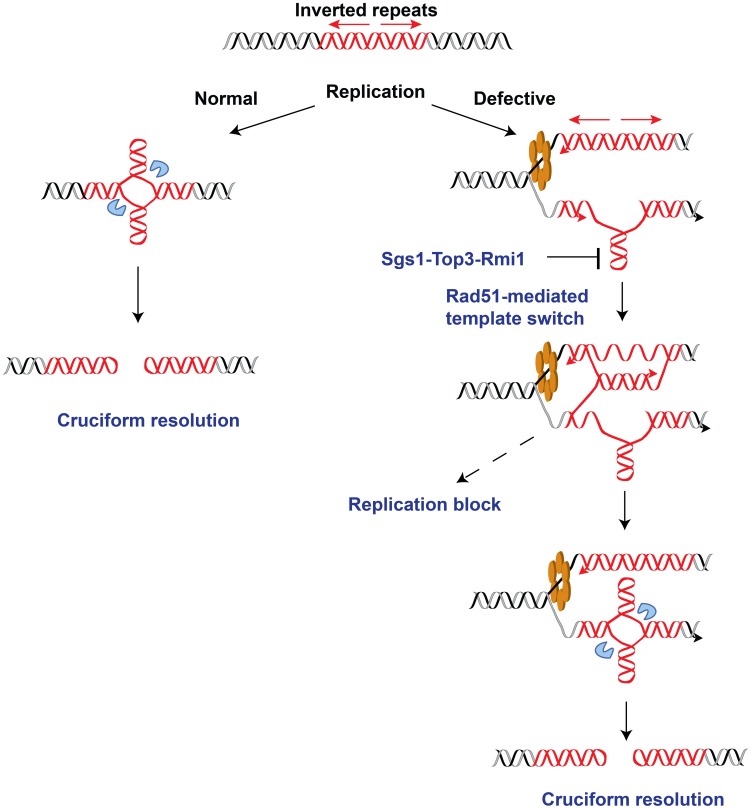
Model for *Alu*-IRs-mediated fragility under conditions of replication proficiency and deficiency. The red helixes, blue pacman and orange hexamer depict the inverted repeats, the putative nuclease and the DNA replication helicase, respectively. In the case of normal replication, cruciform structure might form outside of S-phase as a result of chromatin packing or remodeling. On the other hand, long single-stranded DNA exposed due to compromised replication would facilitate the formation of a hairpin, which could further be converted into cruciform structure via template switch by Rad51. The intermediates of template switching present a strong obstacle for the replication machinery that is manifested as replication block in the replication-deficient strains. The Sgs1-Top3-Rmi1 dissolvosome might participate in unwinding the hairpin. Once formed, the cruciform structure might be attacked by the putative nuclease, leading to DSBs at the IRs.

To gain better insight into the mechanism underlying *Alu*-IR-induced fragility, we monitored replication progression through 100% homologous repeats in the wild-type strain and the replication-deficient mutant TET-*POL3* using 2D gel electrophoresis and Southern hybridization. While replication progression was not hampered at the quasi-palindrome in the wild-type strain, the TET-*POL3* mutant demonstrated a robust fork arrest at the repeats. The fact that the replication block in TET-*POL3* is completely removed upon deletion of *RAD51* (Figure 5) argues for Rad51-mediated template switching as the signal for replication pausing. These data, along with the observation that Δ*rad51* suppresses DSB formation in replication deficient strains, support the scenario where an attempt to bypass hairpin structures during compromised replication via Rad51-dependent template switching promotes the formation of cruciform structures behind the replication fork. These structures are further attacked by nucleases, resulting in DSBs (Figure 6). Although DSB formation in other hyper-fragile mutants in Δ*rad51* background was not analyzed, the fact that the GCR levels in these strains decreased as compared to their *RAD51* counterparts strongly suggests that the same mechanism of break formation operates in these mutants (Table S2).

Overall, these data reveal an important role of homologous recombination in promoting DSB formation at inverted repeats, specifically in replication-deficient mutants.

## Discussion

Palindromic sequences are strong inducers of DSBs and rearrangements in both prokaryotes and eukaryotes. The two distinct events that trigger fragility are considered to be the formation of either hairpin or cruciform structures at the repeats. In this study we found that when replication is compromised, replication delay imposed by inverted repeats is channeled into cruciform resolution via the action of homologous recombination pathway. These data led us to propose that the transition from hairpin to cruciform formation through Rad51-mediated template switching is the mechanism for fragility operating in cells under replication stress.

### Genome-wide screen identifies intact replication as a major guardian of quasi-palindrome stability

Inverted repeat-induced GCRs can be augmented in mutants that either influence secondary structure metabolism or alter repair of the broken chromosome. Previous studies from our lab have demonstrated that *Alu*-IRs-induced DSBs have hairpin termini that are opened by the Mre11 complex and Sae2 to initiate resection [5]. Unprocessed hairpin-capped molecules lead to the formation of acentric and dicentric inverted chromosomes. Detailed analysis of GCR events showed that dicentric chromosomes are stabilized as a result of breakage in anaphase, followed by resection and repair preferentially via break-induced replication with non-homologous chromosomes. It is important to note that DSB resection that precedes the healing of the broken chromosome activates checkpoint signaling and is manifested as cells arrested in G2/M [6]. Previously, we found that GCR rates are elevated in *mrx* mutants. This increase is not due to frequent DSBs at *Alu*-IRs, but rather a result of more efficient formation of dicentric chromosomes and loss of the broken acentric fragments. Consistently, Δ*mre11*, Δ*rad50*, and Δ*xrs2* were identified in this genome-wide screen as hyper-fragile mutants (Table 1 and Table S1). Another group of mutants that do not increase breakage but amplify GCR rates are those defective in DNA damage checkpoint signaling (Δ*rad17*, Δ*mec3*, Δ*ddc1*, Δ*rad24*) (Table 1, Table S1 and Figure S2). It is conceivable that in the absence of checkpoint activation after dicentric breakage, the rate of resection is decreased [55] and the broken chromosomes are replicated and segregated together to the daughter cells for several generations [56], [57], which improves their chances for repair.

The mutants identified in the screen that increase DSB formation and GCRs at *Alu*-IRs are deficient in DNA replication, replication-pausing checkpoint surveillance, Fe-S cluster biogenesis, telomere maintenance and protection, or the function of the Sgs1-Rmi1-Top3 dissolvasome. As discussed below, the impact of deficiencies in these different processes on fragility can be explained by an increase in the probability of formation or stability of secondary structures during replication.

The screen revealed that depletion of the major components of the replication fork responsible for synthesis of both leading and lagging strands increases *Alu*-IR-induced fragility. Our results are consistent with previous findings that mutations in the DNA polymerases α and δ promote excision of IRs and IRs-induced recombination and rearrangements [2], [3], [35], [38], [58]. It is possible that deficiencies in the synthesis of either the leading or lagging strand can lead to the generation of extensive single-stranded regions, thereby creating ideal conditions for the formation of hairpin structure, the initial event in *Alu*-IRs fragility (Figure 6). In replication-proficient mutants mismatches strongly suppress the fragility potential of inverted repeats which should be expected if cruciform extrusion is the initial step in breakage. However, in replication-deficient strains where transient hairpin structure probably precedes cruciform formation, mismatches in the inverted repeats are expected to have a lower impact on the formation of hairpin structure due to the presence of single stranded regions. This might explain the higher relative increase in fragility at imperfect repeats in comparison with repeats without heterology, for example in TET-*RFA2*, TET-*POL2* and TET-*PRI1* mutants (Table 1). Interestingly, downregulation of the helicase Mcm2-7 and ORC also led to hyper-fragility at the repeats. Although the MCM helicase is a part of the replication machinery, it travels ahead of the fork, therefore generation of ssDNA due to depletion of this helicase is unlikely. The effect of deficiencies in MCMs and ORC on fragility might be the consequence of the inability of the closest origin (*ARS*507) to fire since amounts of both protein complexes are important for regulating the timing of origin activation [59]. Replication forks traveling longer distances from the remote origins might be less processive and more prone to collapse upon encountering replication barriers. Downregulation of MCMs and ORC also increases instability at another fragile motif in yeast, the triplex-forming GAA/TTC repeats [43], indicating that this phenomenon might be universal in situations when the replication fork passes through difficult regions. Consistent with this assertion, in human cell lines that have different replication landscapes, fragility at FRA3B and FRA16D sites depends on the distance the replication fork travels [60]. Alternatively, increased fragility in mutants for MCMs and ORC might be due to the assembly of a hampered replisome that lacks components required for leading or lagging strand synthesis.

Deletion of *MMS19* and downregulation of *YHR122W*, genes encoding proteins involved in Fe-S cluster biogenesis [48], [49], were also found to induce hyper-fragility at *Alu*-IRs. Recently, it has been shown that Mms19 and Yhr122W along with Cia1, are required for the transfer of Fe-S clusters to various proteins including polymerase δ DNA primase and Dna2 [48], [49], deficiencies in which were identified to augment fragility in the screen. The presence of the Fe-S clusters in the polymerases α and ε [61] and the fact that these proteins interact with Mms19 [48] also makes them likely substrates for the CIA targeting complex. The effect of mutation in this pathway on *Alu*-IRs-mediated fragility can therefore be attributed to the impaired maturation of the DNA replication machinery.

The deficiencies described above are expected to create optimal conditions for the formation of a hairpin that impedes replication progression. The hairpin might be formed at lower frequencies in replication-proficient cells as well. In both replication-proficient and -deficient strains, the secondary structure or the arrested fork might trigger the activation of checkpoint response required to recruit proteins to remove the hairpin and promote replication restart (Figure 6). The fact that deficiency of Mec1 and the Mrc1-Tof1-Csm3 complex leads to hyper-fragility implicates these proteins as possible sensors of secondary-structure-imposed replication arrest. However, the Mrc1-Tof1-Csm3 complex is also required to coordinate the Mcm2-7 helicase and DNA polymerase activities [53], [62]–[64], therefore, we cannot completely rule out that deficiencies in this complex affect the integrity of the replisome as well.

It seemed reasonable to suggest the existence of helicases recruited to remove hairpins at the arrested fork. Indeed, the screen identified the Sgs1-Top3-Rmi1dissolvasome. Although Δ*sgs1* does not affect the stability of short CAG/CTG repeats (less than 25 repeats), it increases the contraction and fragility rate of long CAG/CTG repeats (70 repeats), indicating that longer hairpins might be better substrates for Sgs1 activity [50], [65]. In addition, the Sgs1-Top3-Rmi1 complex is involved in the dissolution of double Holliday junctions [66]. Hence, it is probable that this complex also irons out long hairpins formed by *Alu*-IRs during replication.

An interesting group of mutants that destabilize *Alu*-IRs include TET-*TEN1*, TET-*CDC13*, and TET-*STN1*. The Cdc13-Stn1-Ten1 (CST) complex is involved in protection of chromosome ends, telomerase recruitment and telomere replication. Hyper-fragility at inverted repeats due to deficiencies in this complex can be explained by the sequestration of the Tof1-Mrc1-Csm3 complex from the replisome to the single-stranded regions at uncapped telomeres [67], [68]. Alternatively, this complex which is structurally similar to RPA [69] may facilitate replication progression through the hairpin. Dewar and Lydall, (2012) proposed that in mammalian cells the CST complex which is distributed throughout the genome [70], aside from its role in telomere metabolism, facilitates replication through difficult regions. Taking into account that downregulation of the CST complex also increases GAA/TTC-mediated fragility and expansions [43] and the physical interaction of this complex with Polα [71], [72], it is reasonable to suggest that the role of CST in DNA replication might be evolutionarily conserved.

### Analysis of DSB intermediates in replication-deficient mutants points towards cruciform-resolution mechanism of fragility

In wild-type strains carrying inverted repeats, the deduced mechanism of breakage is cruciform-resolution by a putative nuclease that cuts symmetrically at the base of the two hairpins. This generates two hairpin-capped molecules that are present in equimolar ratios [5]. Since replication is a polar process, in replication-deficient strains, a nuclease attack on the accumulated hairpins or stalled replication fork would be expected to produce DSB intermediates different from those induced in the wild-type strains. Anticipated intermediates would include nicked hairpins, branched structures, or asymmetrical hairpin-capped breaks. Somewhat unexpectedly, we found that in the TET-*POL3* strain in the absence of Sae2, the DSB intermediates were structurally identical to the replication-proficient strains: only covalently-closed hairpin-capped breaks and inverted dimers resulting from replication of the DSBs were detected. Accumulation of hairpin-capped intermediates on both sides of the break indicates that cruciform-resolution is the predominant pathway for fragility under replication stress. Since deletion of *SAE2* leads to stabilization of hairpin-capped breaks in all mutants analyzed, we propose that this mechanism operates not only in the TET-*POL3* strain, but also in other hyper-GCR mutants identified in the screen.

### Homologous recombination machinery is a key mediator of fragility in replication-deficient mutants

Based on our finding that deletion of *RAD51* or *RAD54* strongly decreases GCRs and breaks in replication-deficient strains (Table 2, Figure 4, Table S2 and Figure S5), we proposed that cruciform formation and resolution can result from the action of the homologous recombination machinery on intermediates present at the stalled replication fork. Consistent with this conjecture, replication arrest observed in TET-*POL3* was also dependent on Rad51. We cannot completely rule out the possibility that homologous recombination proteins facilitate the hairpin formation. However, taking into account that Rad51 forms nucleoprotein filaments that are essential for the invasion step of homologous recombination and that Rad54 promotes strand exchange [73], we favor the explanation that Rad51 along with other components of the homologous recombination machinery promotes template switching when the replication fork encounters the hairpin structure. Synthesis of the hairpin-forming sequence on the unperturbed strand and reannealing of this newly synthesized DNA might allow formation of a cruciform structure which is resolved by a putative nuclease to give rise to hairpin-capped DSBs (Figure 6). In this case, the replication stalling observed in TET-*POL3* would reflect the accumulation of arrested forks in response to template switching rather than inhibition of DNA synthesis by the hairpin structure. Rad51 was found to be present at unperturbed and stalled replication forks [74]–[77], and the involvement of recombination proteins in the fork restart and bypass of DNA lesions via template switching has been demonstrated in several studies [78]–[84]. Here, we show that the attempt of homologous recombination proteins to bypass the secondary-structure barrier may be detrimental and culminate in breaks and GCRs. We also observed that in the TET-*POL2* mutant there was a stronger Rad51-dependent increase in dimer formation than in the accumulation of DSBs indicating that at least in the situation where the leading strand synthesis is compromised template switch might culminate in the inversion of the replication fork. This pathway previously described in *S. pombe* [80], [81], that does not generate DSBs might operate in parallel with the cruciform resolution pathway.

It is important to note that the Rad51 effect is specific in situations where replication is compromised. In replication-proficient strains, breaks and GCRs are not affected by Rad51 status, indicating that another mechanism for cruciform-formation exists. It is possible that in wild-type strains a homologous recombination-independent template switching mechanism leading to fragility operates, or that the cruciform formation is unrelated to replication. The latter hypothesis is supported by our recent finding that hairpin-capped breaks in the wild-type strain preferentially occur in G2 phase of the cell cycle (Sheng *et al.*, in preparation).

Based on this study, we propose that in the human population, the carriers of hypomorphic alleles for the BLM-hTOPOIIIα-hRMI1-hRMI2 dissolvasome and proteins involved in DNA replication, replication-pausing checkpoint surveillance, Fe-S cluster biogenesis, telomere maintenance and protection might be susceptible to inverted repeat-induced breaks and carcinogenic GCRs. Importantly, the status of these proteins determines the stability of imperfect repeats with a spacer and divergent arms that are present in the human genome [41], [85]. At the same time, it is likely that homologous recombination can trigger chromosomal breakage at secondary structure-forming fragile sites and AT-rich palindromic sequences under conditions of replication stress. This detrimental role of homologous recombination in promoting chromosomal instability might contribute towards the development of diseases associated with fragile motifs. Homologous recombination-mediated chromosomal breakage and rearrangements might operate at secondary structure-forming fragile sites and AT-rich palindromic sequences under replication stress. This detrimental role of homologous recombination in promoting genome instability might contribute towards the development of diseases.

## Materials and Methods

### Yeast strains

yTHC, DAmP and YKO collections were purchased from Open Biosystems. All other strains in this study are derivatives of BY4742 (Open Biosystems). The genotype of the query strains for the screen is: MATα, Δ*ura3*, Δ*leu2*, Δ*his3*, Δ*lys2*, *rpl28-Q38K*, Δ *mfa1::MFA1pr-HIS3*, *V34205::lys2::Alu*-IRs, *V29617::hphMX*. The 100% or 94% homologous inverted *Alus* were inserted into the *LYS2* gene via the pop-in and pop-out method as previously described [9]. The detailed construction of the query strain can be found in Zhang et al., 2012 [43].

The effect of mutant alleles identified from the screen was verified in derivatives of YKL36 that carries the GCR assay and has the following genotype: MATa, Δ*bar1*, Δ*trp1*, Δ*his3*, Δ*ura3*, Δ*leu2*, Δ*ade2*, Δ*lys2*, *V34205*::*ADE2*, *lys2*::*Alu*-IRs. To create the mutant strains, in the case of non-essential genes, the target gene was disrupted by the *kanMX4* cassette [86]; in the case of essential genes, the repressible *tetO7* promoter construct was PCR-amplified [44] from pCM225 (Euroscarf) and was used to replace the natural promoter of the gene to create the TET-alleles (Table S3).

In strains used for DSB analysis, *SAE2* was disrupted by *TRP1*. For construction of the Δ*sgs1*Δ*hdf1*Δ*sae2* triple mutant, *SGS1* was disrupted by the *kanMX4 cassette*, and *HDF1* was knocked out by the *hphMX* cassette [87] (Table S3). To study the effect of *RAD51* on *Alu*-IRs-mediated fragility, *RAD51* was replaced by a hisG-*URA3*-hisG cassette [88].

### Genome-wide screen scheme

The screen was carried out as described in Zhang et al., 2012 [43].

### Measurement of GCR rates

Yeast cells were grown on YPD plates for 3 days. For each strain, a minimum of 14 independent colonies were taken to perform fluctuation test to estimate GCR rates. Appropriate dilutions of cells were plated on YPD and canavanine-containing plates to determine the GCR frequency. The GCR rates were calculated using the formula μ = f/ln(Nμ) as described in Drake, 1991 [89]. 95% confidence intervals were calculated as described in Dixon, 1969 [90]. The canavanine-containing plates used for tests were made from arginine-drop out medium with low amount of adenine (5 mg/L) and supplemented with L-canavanine (60 mg/L).

### DSB detection

Yeast cells from overnight cultures were embedded into 0.8% low-melting agarose plugs at a concentration of 24×10^8^ cells/ml. The plugs were treated with 1.5 mg/ml lyticase for 3 hr, followed by overnight 1 mg/ml proteinase K treatment. For restriction digestion of the DNA, the plugs were washed twice with 1 X TE buffer (10 mM Tris-Cl [pH 8.0], 0.1 mM EDTA) for 30 min, treated with 1 mM PMSF for 1 hr, washed with distilled water for 1 hr and equilibrated with restriction buffer for 20 min. Each plug (∼40 µl) was digested with 50 units of AflII or BglII for 16 hr. Digested plugs were loaded in a 1% (AflII digestion) or 0.7% (BglII digestion) agarose gel, respectively, and run in 1 X TBE for 18 hr. The gels were treated with 0.25 N HCl for 20 min, alkaline buffer (1.5 M NaCl, 0.5 M NaOH) for 30 min and neutralization buffer (1.5 M NaCl, 1 M Tris [pH 7.5]) for 30 min. The gels were then transferred in 10 X SSC to charged nylon membrane for 2 hr through a Posiblotter (Stratagene). Southern hybridization was carried out using P^32^-labeled *LYS2*-specific probes at 67°C overnight. DNA membranes were washed twice for 15 min each in buffer containing 0.1% SDS and 1% SSC and the signals were detected by the typhoon phosphoimager (GE Healthcare Life Sciences). The hybridization signals were quantified using ImageJ software (NIH).

### 2D neutral/neutral and neutral/alkaline gels for analyzing the structure of the broken ends

Yeast plugs were prepared and digested as described above. Neutral/neutral and neutral/alkaline gel analysis was performed as previously described with small modifications [5], [91]. In the first dimensional gel electrophoresis, the plugs were loaded in a 1% (AflII digestion) or 0.7% agarose (BglII digestion) gel, respectively, and run for 18 hr in 1 X TBE. The gel slices containing the bands of interest were then cut out for the second dimensional gel electrophoresis. For neutral/neutral gel analysis, the gel slices were loaded in 1% (AflII digestion) or 0.7% (BglII digestion) agarose gel made in 1 X TBE, run in 1 X TBE for 18 hr at 1.7 V/cm and then processed for Southern hybridization. For neutral/alkaline gel, the gel slices were treated with 10 mM EDTA for 30 min, 5 mM EDTA for 30 min and embedded in agarose gel made in buffer containing 50 mM NaCl, 1 mM EDTA. Next, the gels were soaked in 5 X alkaline buffer for 30 min, 1 X alkaline buffer (50 mM NaOH, 1 mM EDTA) for 30 min and cooled down in 1 X alkaline buffer at 4°C for 15 min. The gels were then run in 1 X alkaline buffer at 0.7 V/cm for 40 hr at 4°C and processed for Southern hybridization.

### 2D neutral/neutral gel analysis for replication fork progression

2D gel analysis was carried out as previously described in Brewer and Fangman, 1987 [92]. Overnight yeast cultures were synchronized in G1 with alpha factor (50 µg/10^7^ cells) at OD600 = 0.8. 2 µg/ml doxycycline was added to the cultures to downregulate Polδ in the case of TET-*POL3* and TET-*POL3*Δ*rad51* strains. Cells were then released into fresh YPD. 50 min after release, wild-type, TET-*POL3*, TET-*POL3*Δ*rad51* strains were harvested and their genomic DNA samples were prepared as described in Friedman and Brewer, 1995 [93]. For the first dimensional gel electrophoresis, AflII digested DNA samples were loaded in a 0.4% agarose gel and run in 1 X TBE at 1.7 V/cm for 22 hr. For the second dimensional gel electrophoresis, gel slices containing bands of interest were cut out and loaded into a 1.2% agarose gel supplemented with 0.3 mg/ml ethidium bromide. The gels were run in 1 X TBE at 6 V/cm for 11 hr. Gels were then processed for Southern hybridization. Images were quantified using ImageQuant TL software (GE Healthcare Life Sciences).

## Supporting Information

Figure S1 The genome-wide screen scheme. In the query strains, the chromosomal arm containing the GCR assay was marked by the *hphMX* cassette. The strains also carried a mating-type-regulated reporter MFApr-*HIS3* and a Q38K mutation in *RPL28* that rendered the strains resistant to cycloheximide. Both modifications serve as selection markers for the haploid strains during the screen. The tester strains were labeled with the *kanMX* cassette and consisted of three libraries: yTHC, DAmP and YKO (Open Biosystems). Each tester strain was crossed with duplicates of the query strains on YPD. The diploids were selected on medium supplemented with G418 and hygromycin and induced for sporulation. Haploid progeny (MATa) were selected on histidine drop-out medium supplemented with cycloheximide. Haploids containing both the repeats and the mutation of interest were selected by G418- and hygromycin-containing medium. The strains were then replica plated to canavanine-containing medium to select for GCR events. For the yTHC library, doxycycline down-regulation (2 µg/ml) of the mutated alleles was applied prior to canavanine selection.
(TIF)

Figure S2 Detection of breakage intermediates in a subset of hyper-GCR mutants. Genomic DNA embedded in agarose plugs were digested by AflII (**A**) or BglII (**B**) and processed for Southern hybridization as described in Figure 2. Strains included in the analysis are: wild-type, Δ*sae2*, Δ*rad17*, Δ*rad17*Δ*sae2*, Δ*mms19*, Δ*mms19*Δ*sae2*, TET-*TEN1*, TET-*TEN1*Δ*sae2*. Bands corresponding to the unbroken fragment, dimer and DSB fragment are indicated by arrows. The star indicates the bands below the unbroken fragment in the Δ*mms19* and Δ*mms19*Δ*sae2* strains, which likely result from partial excision of the inverted repeats in these strains. (**C**) and (**D**) Densitometry analysis of the broken fragments normalized to the intact chromosome V in Δ*sae2* strains in (**A**) and (**B**), respectively. Values are shown as mean (shown on the top of the bars) with standard deviation obtained from at least three independent experiments.
(TIF)

Figure S3 Detection of breakage intermediates in Δ*hdf1* and wild-type strains. (**A**) Genomic DNA for wild-type, Δ*sae2*, Δ*hdf1* and Δ*hdf1*Δ*sae2* were embedded in agarose plugs and digested by AflII and processed for Southern hybridization as described in Figure 2. Bands corresponding to the unbroken fragment, dimer and DSB fragment are indicated by arrows. (**B**) Densitometry analysis of the broken fragments normalized to the intact chromosome V in Δ*sae2* strains. Values are shown as mean (shown on the top of the bars) with standard deviation obtained from at least three independent experiments.
(TIF)

Figure S4 Rad51 dependent breakage formation in Δ*mec1* mutants. (**A**) DSB intermediates for the strains Δ*sae2*, Δ*sml1*Δ*sae2*, Δ*sml1*Δ*mec1*Δ*sae2* and Δ*sml1*Δ*mec1*Δ*rad51*Δ*sae2* were detected using AflII digestion and Southern hybridization as described in Figure 2. The unbroken fragment, dimer and DSB fragment are indicated by arrows. (**B**) Densitometry analysis of the broken fragments normalized to the intact chromosome V in Δ*sae2* strains. Values are shown as mean (shown on the top of the bars) with standard deviation obtained from at least three independent experiments.
(TIF)

Figure S5 DSB accumulation in TET-*POL3* but not in wild-type strains is dependent on Rad54. (**A**) Southern analysis was performed for the breakage intermediates in Δ*sae2*, Δ*rad54*Δ*sae2*, TET-*POL3*Δ*sae2* and TET-*POL3*Δ*rad54*Δ*sae2* mutants upon digestion of genomic embedded in agarose plugs by AflII. Arrows indicate the unbroken fragment, dimer and DSB fragment. (**B**) Densitometry analysis of the broken fragments normalized to the intact chromosome V in Δ*sae2* strains. Values are shown as mean (shown on the top of the bars) with standard deviation obtained from at least three independent experiments.
(TIF)

Table S1 Hyper-GCR mutants identified in the genome-wide screen. ^a^+ shows mutants identified as hyper-GCR alleles from the libraries indicated. ^b^* shows mutant alleles whose effect on *Alu*-IR- mediated GCRs were determined by fluctuation tests.
(DOCX)

Table S2 Effect of *RAD51* and *RAD54* deletion on *Alu*-IR-mediated GCR in mutants identified from the screen. ^a^ Numbers in the brackets are 95% confidence intervals of the fluctuation tests.
(DOCX)

Table S3 Primers used in the study.
(DOCX)
