# Supplementary figures and images for: Genome-Wide Screen Reveals Replication Pathway for Quasi-Palindrome Fragility Dependent on Homologous Recombination
Source: PLoS Genet. 2013 Dec 5;9(12):e1003979. doi: 10.1371/journal.pgen.1003979 (PMC3855049; doi:10.1371/journal.pgen.1003979)

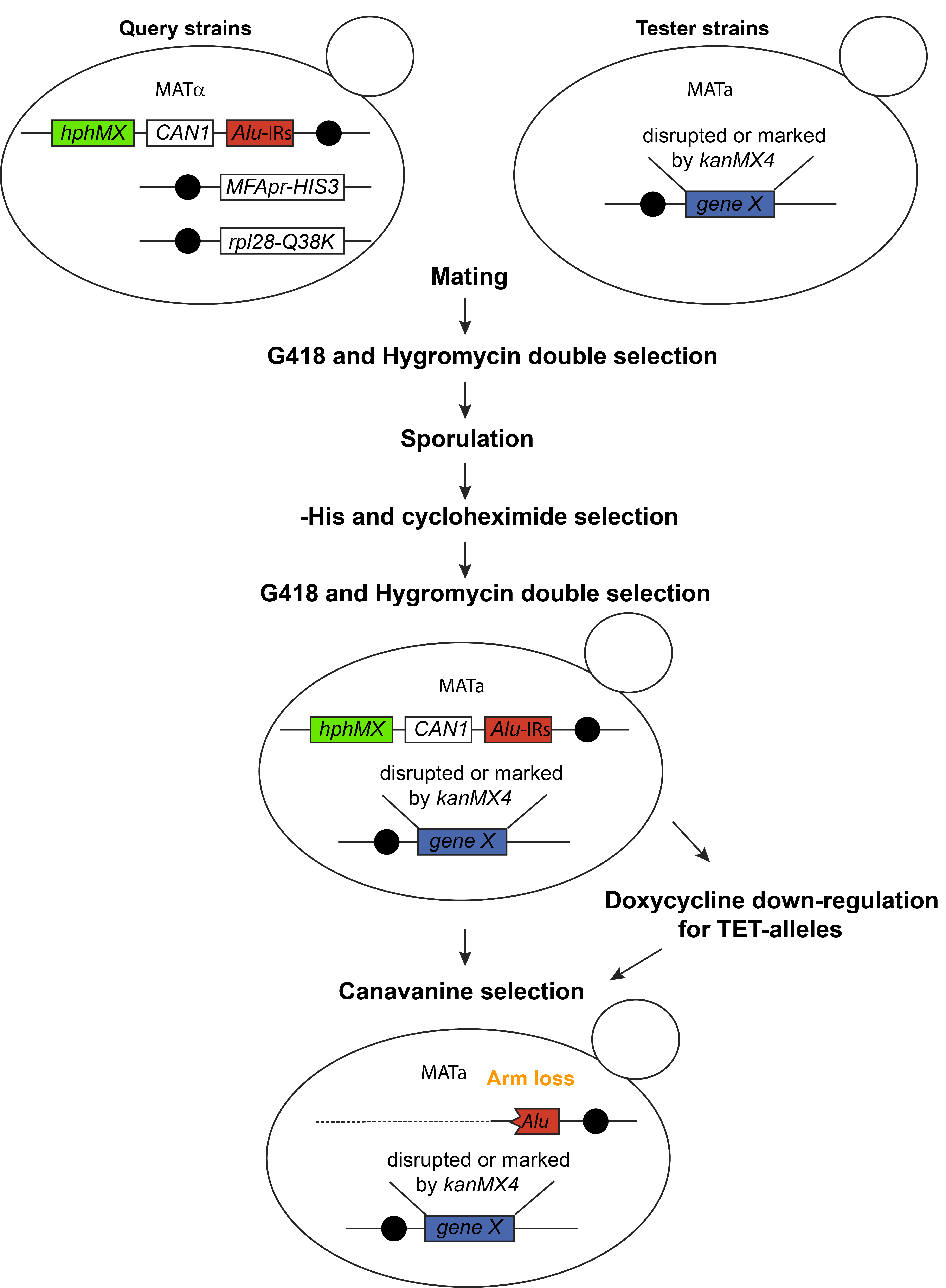

Supplement: Figure S1 — The genome-wide screen scheme. In the query strains, the chromosomal arm containing the GCR assay was marked by the hphMX cassette. The strains also carried a mating-type-regulated reporter MFApr-HIS3 and a Q38K mutation in RPL28 that rendered the strains resistant to cycloheximide. Both modifications serve as selection markers for the haploid strains during the screen. The tester strains were labeled with the kanMX cassette and consisted of three libraries: yTHC, DAmP and YKO (Open Biosystems). Each tester strain was crossed with duplicates of the query strains on YPD. The diploids were selected on medium supplemented with G418 and hygromycin and induced for sporulation. Haploid progeny (MATa) were selected on histidine drop-out medium supplemented with cycloheximide. Haploids containing both the repeats and the mutation of interest were selected by G418- and hygromycin-containing medium. The strains were then replica plated to canavanine-containing medium to select for GCR events. For the yTHC library, doxycycline down-regulation (2 µg/ml) of the mutated alleles was applied prior to canavanine selection. (TIF) [file pgen.1003979.s001.tif]

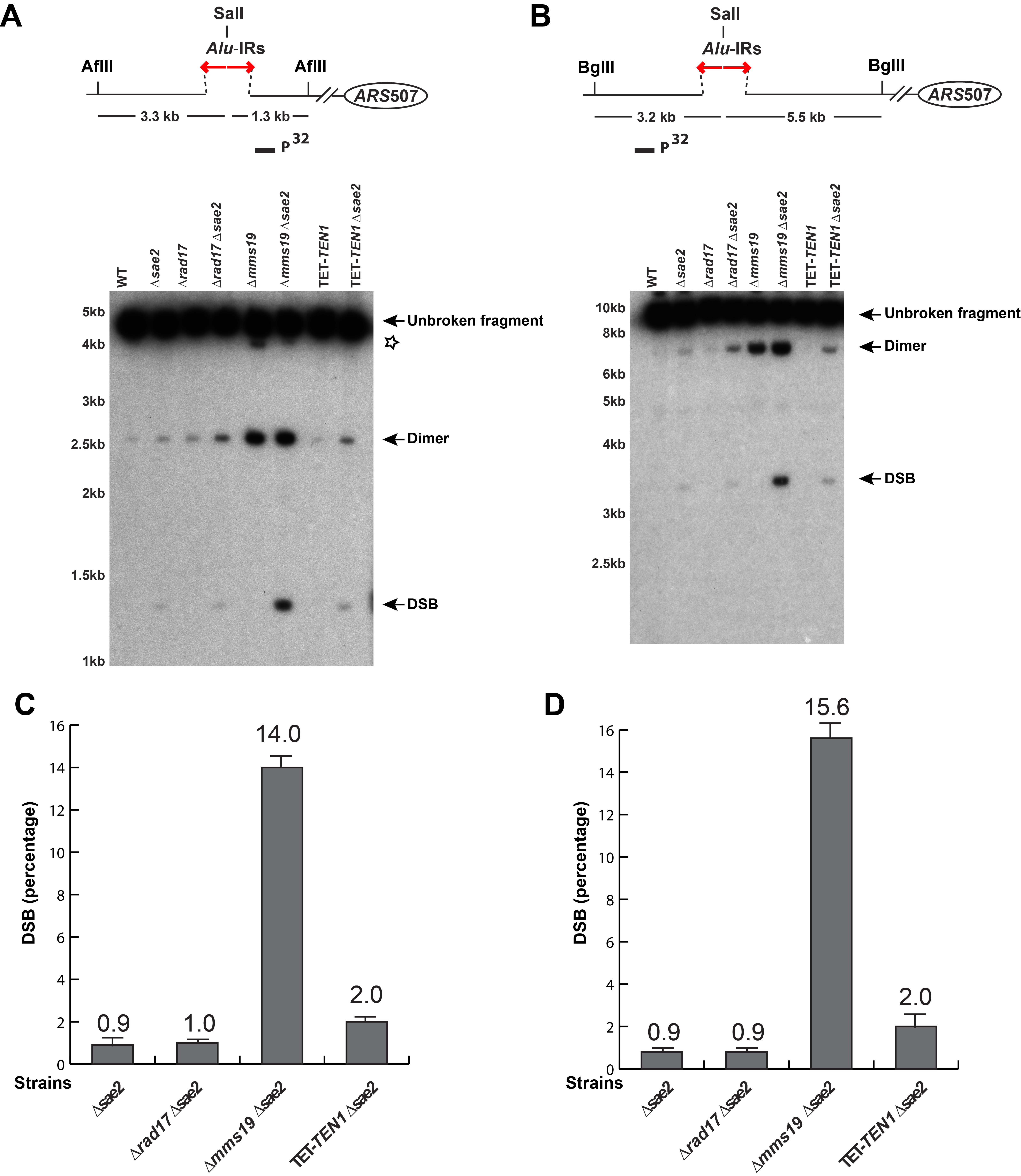

Supplement: Figure S2 — Detection of breakage intermediates in a subset of hyper-GCR mutants. Genomic DNA embedded in agarose plugs were digested by AflII (A) or BglII (B) and processed for Southern hybridization as described in Figure 2. Strains included in the analysis are: wild-type, Δsae2, Δrad17, Δrad17Δsae2, Δmms19, Δmms19Δsae2, TET-TEN1, TET-TEN1Δsae2. Bands corresponding to the unbroken fragment, dimer and DSB fragment are indicated by arrows. The star indicates the bands below the unbroken fragment in the Δmms19 and Δmms19Δsae2 strains, which likely result from partial excision of the inverted repeats in these strains. (C) and (D) Densitometry analysis of the broken fragments normalized to the intact chromosome V in Δsae2 strains in (A) and (B), respectively. Values are shown as mean (shown on the top of the bars) with standard deviation obtained from at least three independent experiments. (TIF) [file pgen.1003979.s002.tif]

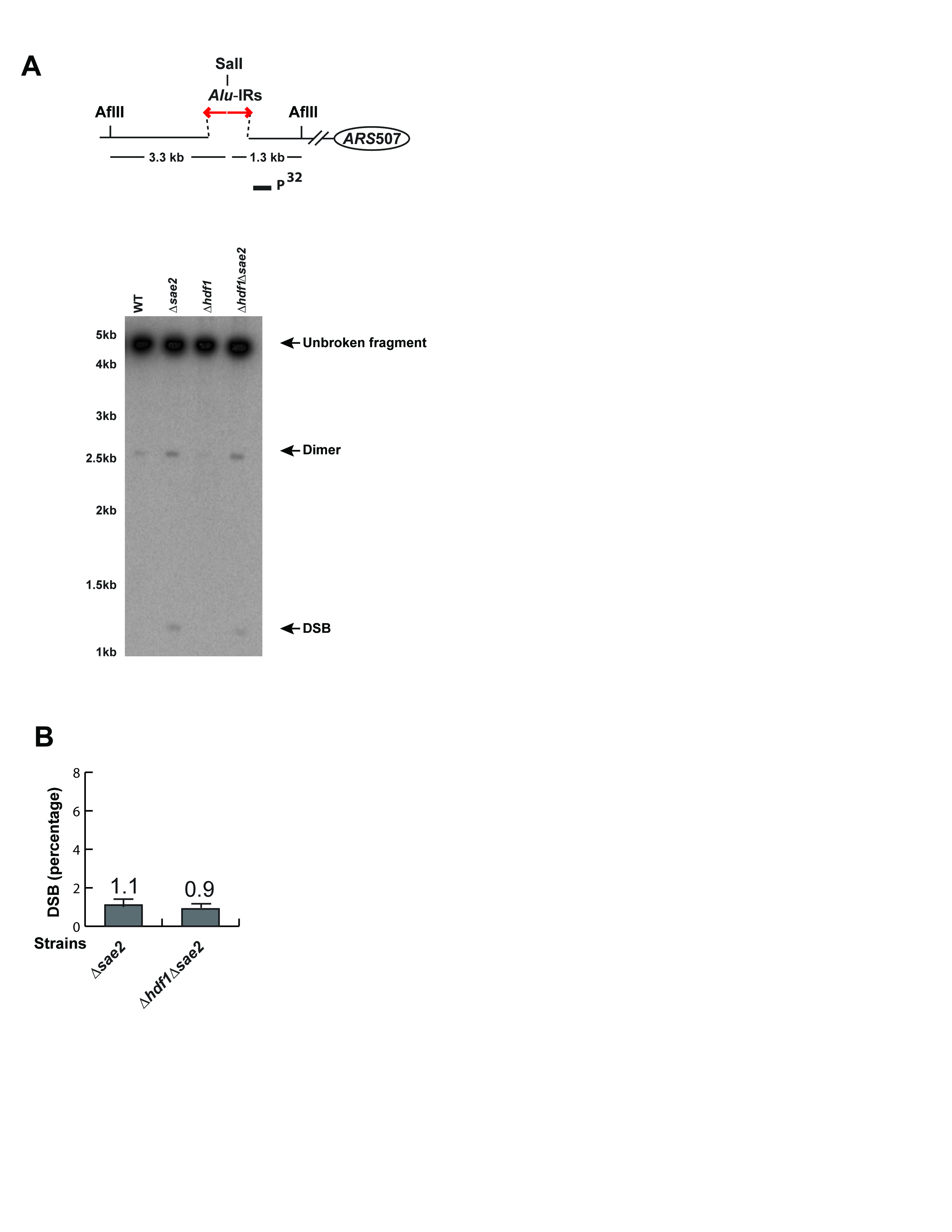

Supplement: Figure S3 — Detection of breakage intermediates in Δhdf1 and wild-type strains. (A) Genomic DNA for wild-type, Δsae2, Δhdf1 and Δhdf1Δsae2 were embedded in agarose plugs and digested by AflII and processed for Southern hybridization as described in Figure 2. Bands corresponding to the unbroken fragment, dimer and DSB fragment are indicated by arrows. (B) Densitometry analysis of the broken fragments normalized to the intact chromosome V in Δsae2 strains. Values are shown as mean (shown on the top of the bars) with standard deviation obtained from at least three independent experiments. (TIF) [file pgen.1003979.s003.tif]

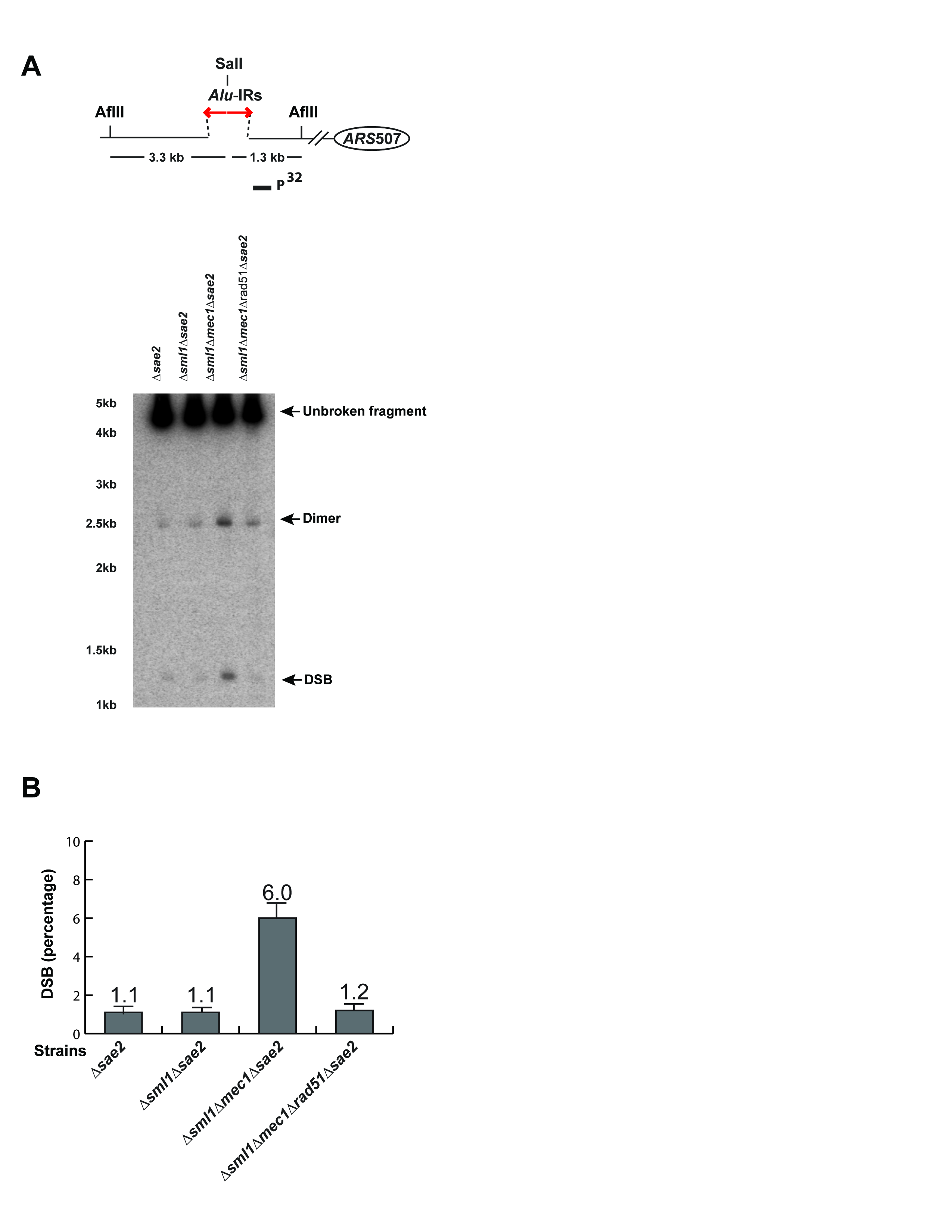

Supplement: Figure S4 — Rad51 dependent breakage formation in Δmec1 mutants. (A) DSB intermediates for the strains Δsae2, Δsml1Δsae2, Δsml1Δmec1Δsae2 and Δsml1Δmec1Δrad51Δsae2 were detected using AflII digestion and Southern hybridization as described in Figure 2. The unbroken fragment, dimer and DSB fragment are indicated by arrows. (B) Densitometry analysis of the broken fragments normalized to the intact chromosome V in Δsae2 strains. Values are shown as mean (shown on the top of the bars) with standard deviation obtained from at least three independent experiments. (TIF) [file pgen.1003979.s004.tif]

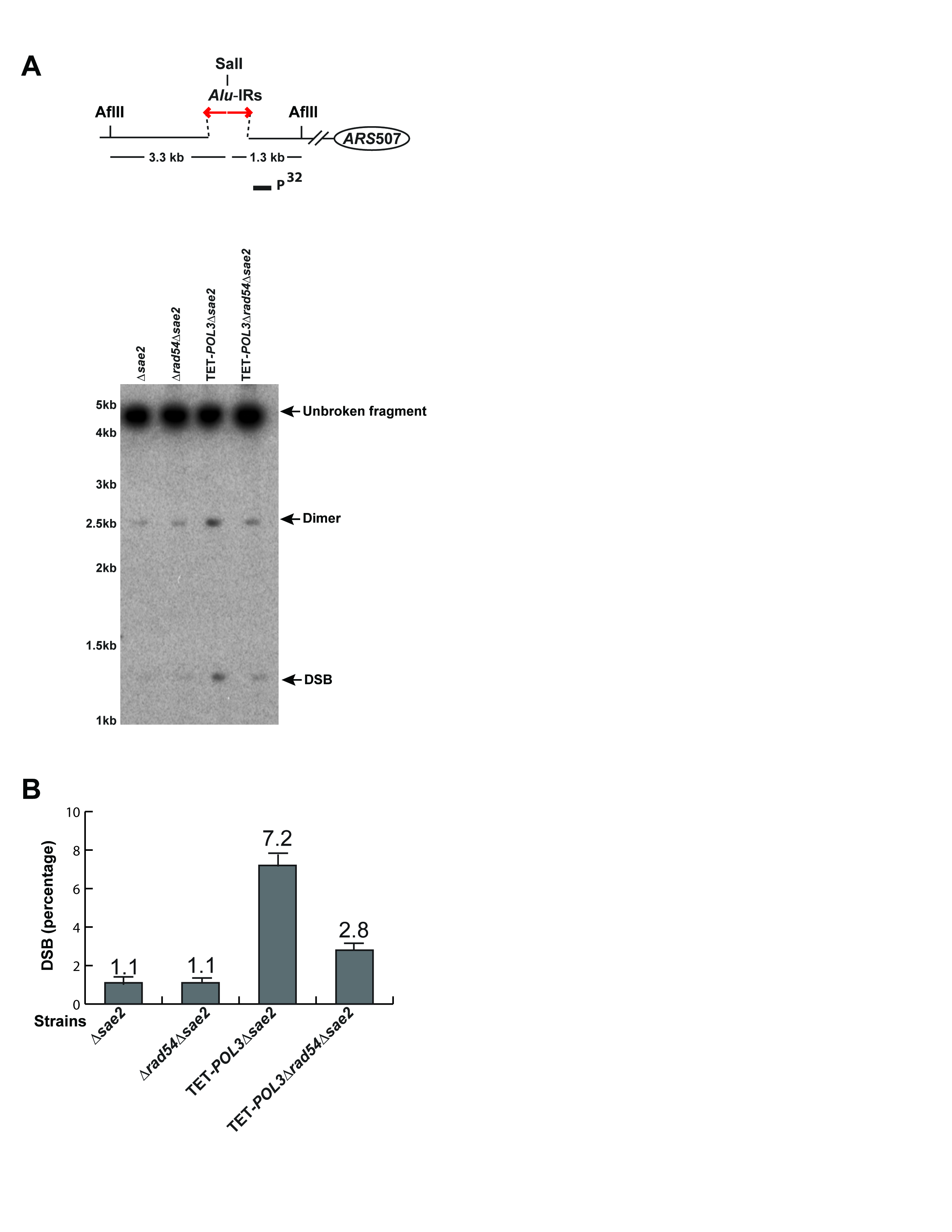

Supplement: Figure S5 — DSB accumulation in TET-POL3 but not in wild-type strains is dependent on Rad54. (A) Southern analysis was performed for the breakage intermediates in Δsae2, Δrad54Δsae2, TET-POL3Δsae2 and TET-POL3Δrad54Δsae2 mutants upon digestion of genomic embedded in agarose plugs by AflII. Arrows indicate the unbroken fragment, dimer and DSB fragment. (B) Densitometry analysis of the broken fragments normalized to the intact chromosome V in Δsae2 strains. Values are shown as mean (shown on the top of the bars) with standard deviation obtained from at least three independent experiments. (TIF) [file pgen.1003979.s005.tif]
