# Supplementary material for: Genome-Wide Screen Reveals Replication Pathway for Quasi-Palindrome Fragility Dependent on Homologous Recombination
Source: PLoS Genet. 2013 Dec 5;9(12):e1003979. doi: 10.1371/journal.pgen.1003979 (PMC3855049; doi:10.1371/journal.pgen.1003979)
Supplement: Table S1 — Hyper-GCR mutants identified in the genome-wide screen. a+ shows mutants identified as hyper-GCR alleles from the libraries indicated. b* shows mutant alleles whose effect on Alu-IR- mediated GCRs were determined by fluctuation tests. (DOCX) [file pgen.1003979.s006.docx]

**Supplemental information**

**Supplemental Tables**

| **Table S1. Hyper-GCR mutants identified in the genome-wide screen** | | | | |
| --- | --- | --- | --- | --- |
| Genetic  background | Essential genes | | Non-essential genes | Mutants recreated |
|  | yTHC | DAmP | YKO |  |
| Double strand breaks repair genes | | | | |
| *mre11* |  |  | +a | *b |
| *rad50* |  |  | + | * |
| *xrs2* |  |  | + |  |
| *sae2* |  |  | + | * |
| Replication genes | | | | |
| *orc2* | + |  |  | * |
| *orc4* | + |  |  | * |
| *mcm4* | + |  |  | * |
| *mcm5* | + |  |  |  |
| *mcm7* | + |  |  |  |
| *rfa2* | + | + |  | * |
| *pol12* | + |  |  |  |
| *pri2* | + |  |  | * |
| *pol1* | + | + |  | * |
| *pol2* |  |  |  | * |
| *pol3* | + |  | + | * |
| *pol32* |  |  |  | * |
| *pol30* | + | + |  | * |
| *rfc2* | + |  |  | * |
| *rfc3* | + |  |  |  |
| *rfc4* | + |  |  |  |
| *rfc5* | + |  |  |  |
| *dna2* | + |  |  | * |
| *rad27* |  |  | + | * |
| *yhr122w* | + |  |  | * |
| *mms19* |  |  |  | * |
| Checkpoint response genes | | | | |
| *tof1* |  |  | + | * |
| *mrc1* |  |  | + |  |
| *csm3* |  |  | + | * |
| *rad17* |  |  | + | * |
| *mec3* |  |  | + |  |
| *ddc1* |  |  | + |  |
| *rad24* |  |  | + | * |
| *dun1* |  |  | + | + |
| Telomere maintenance genes | | | | |
| *ten1* |  | + |  | * |
| *cdc13* |  |  |  | * |
| Sgs1-Top3-Rmi1 dissolvosome | | | | |
| *sgs1* |  |  | + | * |
| *rmi1* |  |  | + |  |
| *ylr235c* |  |  |  |  |

a+ shows mutants identified as hyper-GCR alleles from the libraries indicated.

b* shows mutant alleles whose effect on *Alu*-IR- mediated GCRs were determined by fluctuation tests.
