# Supplementary material for: Genome-Wide Screen Reveals Replication Pathway for Quasi-Palindrome Fragility Dependent on Homologous Recombination
Source: PLoS Genet. 2013 Dec 5;9(12):e1003979. doi: 10.1371/journal.pgen.1003979 (PMC3855049; doi:10.1371/journal.pgen.1003979)
Supplement: Table S2 — Effect of RAD51 and RAD54 deletion on Alu-IR-mediated GCR in mutants identified from the screen. a Numbers in the brackets are 95% confidence intervals of the fluctuation tests. (DOCX) [file pgen.1003979.s007.docx]

**Supplemental information**

**Supplemental Tables**

| **Table S2. Effect of *RAD51* and *RAD54* deletion on *Alu*-IR-mediated GCR in mutants identified from the screen** | | |
| --- | --- | --- |
| Genetic background | GCR rate (X10^-6^) | Fold increase over wild-type |
| WT (100% *Alu*-IRs) | 41 (30-52)a | 1 |
| Δ*rad51* | 37 (27-50) | 1 |
| TET*-POL30* | 370 (290-390) | 9 |
| TET*-Pol30*Δ*rad51* | 67 (46-145) | 1 |
| TET*-POL1* | 470 (380-500) | 11 |
| TET-*POl1*Δ*rad51* | 170 (150-260) | 4 |
| TET*-RFA2* | 250 (100-280) | 6 |
| TET-*RFA2*Δ*rad51* | 92 (65-120) | 2 |
| TET*-MCM2* | 150 (140-240) | 4 |
| TET*-MCM2*Δ*rad51* | 44 (28-71) | 1 |
| TET*-ORC4* | 110 (80-230) | 3 |
| TET*-ORC4*Δ*rad51* | 60 (29-100) | 1 |
| TET*-TEN1* | 140 (120-230) | 3 |
| TET*-TEN1*Δ*rad51* | 40 (32-50) | 1 |
| TET*-yhr122W* | 140 (110-160) | 3 |
| TET*-yhr122W*Δ*rad51* | 48 (39-53) | 1 |
| Δ*csm3* | 370 (270-530) | 9 |
| Δ*csm3*Δ*rad51* | 22 (18-34) | 0.5 |
| Δ*sgs1* | 410 (300-490) | 10 |
| Δ*sgs1*Δ*rad51* | 52 (42-53) | 1 |
| Δ*rad17* | 180 (160-250) | 4 |
| Δ*rad17*Δ*rad51* | 100 (75-100) | 2 |
| Δ*mec1*Δ*sml1* | 200 (160-220) | 5 |
| Δ*mec1*Δ*sml1*Δ*rad51* | 56 (50-59) | 1 |
| Δ*rad54* | 25 (22-36) | 1 |
| TET*-POL3* | 460 (390-640) | 11 |
| TET*-POL3*Δ*rad54* | 48 (42-73) | 1 |

a. Numbers in the brackets are 95% confidence intervals of the fluctuation tests
