# Supplementary material for: Genome-Wide Screen Reveals Replication Pathway for Quasi-Palindrome Fragility Dependent on Homologous Recombination
Source: PLoS Genet. 2013 Dec 5;9(12):e1003979. doi: 10.1371/journal.pgen.1003979 (PMC3855049; doi:10.1371/journal.pgen.1003979)
Supplement: Table S3 — Primers used in the study. (DOCX) [file pgen.1003979.s008.docx]

**Supplemental information**

**Supplemental Tables**

| **Table S3. Primers used in the study** | | |
| --- | --- | --- |
| Primer | Sequence | Purpose |
| *POL3*-TET51 | GACGGTTGATCTTGGTTTTGTGTGGTAAAAGTATGCCTTAACGTAATAGGCAGCTGAAGCTTCGTACGC | To replace the natural promoter of *POL3* with the *tetO7* tetracycline downregulatable promoter |
| *POL3*-TET31 | CTCGTCATCGATCTTCACATCAACCATGGGAAGGGATCTTTTTTCACTCATgcataggccactagtggat |  |
| *POL3*-TET52 | GTTCTCCTTTATATCTGCGTTTCTTTGCAGCGTTCTTGCTTGACGGTTGATCTTGGTTTTG |  |
| *POL3*-TET32 | GATTGCCGTTTGATTTTCTTTTCCAACTGGGGAGTATCCTCGTCATCGATCTTCACATC |  |
| *POL1*-TET51 | CTTGTACTGCCTGCAATCTCTATTCTTCATTCATCACACATCTATTCCAGCTGAAGCTTCGTACGC | To replace the natural promoter of *POL1*  with the *tetO7* tetracycline downregulatable promoter |
| *POL1*-TET31 | GCGGCTTGCAGCTTTCTCAATTTTTCTAATTTTTCACTTTTAGAAGACATgcataggccactagtggat |  |
| *POL1*-TET52 | ACGTGCATTTTTCTTAAAGAAATATAACTTTTTTCTTGTACTGCCTGCAATCTCTATTC |  |
| *POL1*-TET32 | CGTCACCTTCGTAATCATCGATGGATGTACCATTTCGAGCGGCTTGCAGCTTTCTCA |  |
| *POL2*-TET51 | GTTTATTGTCATTTGAATTGTCAGAATGGTTTATTTTCAGGTAGGGTAACCAGCTGAAGCTTCGTACGC | To replace the natural promoter of *POL2* with the *tetO7* tetracycline downregulatable promoter |
| *POL2*-TET31 | CTTGCAGTGGAAGATCCTCCGTTGTTTTTTTTCTTGCCAAACATCATgcataggccactagtggat |  |
| *POL2*-TET52 | GTAATAGTAACACTGTCAGTGTTCGTCAAAGGCCCAAGTTTATTGTCATTTGAATTG |  |
| *POL2*-TET32 | GTGTGTTGTACTTGTTGCCAGCTGAATATCTTGCAGTGGAAGATCCTCCG |  |
| *POL30*-TET51 | GATTCTTCAATATCTAATTATTTAGCATTTTTCTTCTCCATCCGACGCACCCAGCTGAAGCTTCGTACGC | To replace the natural promoter of *POL30* with the *tetO7* tetracycline downregulatable promoter |
| *POL30*-TET31 | CAATTATTCTCTTGAAAAGGGATGCTTCTTCAAATTTTGCTTCTAACATgcataggccactagtggat |  |
| *POL30*-TET52 | CTCTTCCTTTTTCGCACAACTTATGCTGATTCTTCAATATCTAATTATTTAG |  |
| *POL30*-TET32 | CATTGGAAATTGACCAACTGGACACAATCTTTGAAACCATCAATTATTCTCTTGAAAAGG |  |
| *YHR122W*-TET51 | GGGTGTACGGTGTTTGAATAATTGAATTAGATTTAAATGCGAATAAGTGA CAGCTGAAGCTTCGTACGC | To replace the natural promoter of *YHR122W*  with the *tetO7* tetracycline downregulatable promoter |
| *YHR122W*-TET31 | TCTAGTGGGAAGTTGGTTCTCCTCTAAAATGTCGGGATTTTCATTCAAAAACTCAGACATgcataggccactagtggat |  |
| *YHR122W*-TET52 | ACTATAATATTCCACCACTCTCTTCTCAGTCGCAATGCTTGGGTGTACGGTGTTTGAATAATTG |  |
| *YHR122W*-TET32 | TGAACCCGCCTAACAAAAGGTCCTTGGTACTATCTTCTTTTCTAGTGGGAAGTTGGTTCTC |  |
| *RFC2*-TET51 | CATCGTTATTTTTCACTCTTTTACTTCGCGTATTTCAAAGCAATCAGGAA CAGCTGAAGCTTCGTACGC | To replace the natural promoter of *RFC2*  with the *tetO7* tetracycline downregulatable promoter |
| *RFC2*-TET31 | GCTAACTTTGATATCTTACGCTTTTTATTTGGACCAAACCCTTCAAACATgcataggccactagtggat |  |
| *RFC2*-TET52 | CACTGTTCCTTTTTTGTTCTCTTCTATCAAAAACCTTTCTCATCGTTATTTTTCACTCTT |  |
| *RFC2*-TET32 | CTCAACCCAGGGTTGTTGTGCCAATGATTGCTCTGCGGCTAACTTTGATATCTTACG |  |
| *RFA2*-TET51 | GTTTCTTAGATAATTATCGCCATATACGAAACGCGTTAGGAAACGCGTTC CAGCTGAAGCTTCGTACGC | To replace the natural promoter of *RFA2*  with the *tetO7* tetracycline downregulatable promoter |
| *RFA2*-TET31 | GCAGAAAAGAGCAAATCCTCCAATTCCTAGCACTAATACATACTTGCCATgcataggccactagtggat |  |
| *RFA2*-TET52 | CCTCGATGAGCTTCCATTTTCAATTTTCATCTTTTTTTTCACGATGCGAAGTTTCTTAGATAATTATCGC |  |
| *RFA2*-TET32 | GTAATTTTAACATTCGCCAATAAAAATAAAAAAAATGATGCAGAAAAGAGCAAATCCTC |  |
| *ORC4*-TET51 | AGTTTCATACATGTGTTCAAGTGTATTTTTGGATTTATCATTTTTC CAGCTGAAGCTTCGTACGC | To replace the natural promoter of *OCR4* with the *tetO7* tetracycline downregulatable promoter |
| *ORC4*-TET31 | GAGAAGATTGACTTGCGGTGATAGACGAGCTTCGCTTATAGTCATgcataggccactagtggat |  |
| *ORC4*-TET52 | TATAATTAAAAAAAAAATAACCATATATTTTGTATTAAGTTTCATACATGTGTTCAAGTG |  |
| *ORC4*-TET32 | GTCTCCTCTACCTCTTCGTTTGAGTGCCTCTTTATTGGGAGAAGATTGACTTGCGGTG |  |
| *DNA2*-TET51 | CTGATCTTACGCTATTTATGGCAAAACTTGTGTTACATTTTTGAAGATCAGCTGAAGCTTCGTACGC | To replace the natural promoter of *DNA2* with the *tetO7* tetracycline downregulatable promoter |
| *DNA2*-TET31 | CAGATATACTCGCAGACCTCTTGTTCTTCTGTGGCGTTCCGGGCATgcataggccactagtggat |  |
| *DNA2*-TET52 | GCAATTCCGTGCGGCAGAAAGAACTTATATATATTTAACTGATCTTACGCTATTTATGGC |  |
| *DNA2*-TET32 | GTATTATTTCTTTTTCCTCTGTCTTCTTCGCAGGTGAAACAGATATACTCGCAGACCTC |  |
| *PRI2*-TET51 | CATATCTGCTGCTCACTCTATTTGCTTTTTCCTGCTCATTTATAGTTACAGCTGAAGCTTCGTACGC | To replace the natural promoter of *PRI2*  with the *tetO7* tetracycline downregulatable promoter |
| *PRI2*-TET31 | TCTTCCTTGAGGCAATTCTTCTTTTTGACTGCCTGAACAT gcataggccactagtggat |  |
| *PRI2*-TET52 | AAGGTGATACGACAGGTCTCTTCTTGTTTCAATATTGCCTTACATATCTGCTGCTCACTC |  |
| *PRI2*-TET32 | TCCAATTCGCTTTTGACAATATCATCGTATGAACTAAAATTCTTCCTTGAGGCAATTC |  |
| *MCM2*-TET51 | CGCGTGTAATATTATTTATCATATGCCTTTTTTAGGAAATCTAATTACTTCAGCTGAAGCTTCGTACGC | To replace the natural promoter of *MCM2* with the *tetO7* tetracycline downregulatable promoter |
| *MCM2*-TET31 | TCTGAGTCCGAATCATCTTCCTCACGTCTACGTCTTCTATTATCAGACATgcataggccactagtggat |  |
| *CDC13*-TET51 | TGTAAGACAATCTTCATTGTAAATCCGCTGCTGAATAATATGTGGATTATCAGCTGAAGCTTCGTACGC | To replace the natural promoter of *CDC13* with the *tetO7* tetracycline downregulatable promoter |
| *CDC13*-TET31 | AAAATACGATTTTTATGTGGAGGACACTCAGGCTCTTCTAAGGTATCCATgcataggccactagtggat |  |
| *TEN1*-TET51 | AGAAATCTTTTGGGCTTTGCCCGCGCAGGTTCGAGTCCTGCAGTTGTCGTCAGCTGAAGCTTCGTACGC | To replace the natural promoter of *TEN1* with the *tetO7* tetracycline downregulatable promoter |
| *TEN1*-TET31 | GTTGCTATTTTATCCTTCAAACATTTTAAGTCTAGAACTAACTGGCTCATgcataggccactagtggat |  |
| S2870 | CTGACGACTCTCAGTTTACCAATGT | To prepare DSB probe for centromere-distal DSB detection.  Also used to prepare 2D probe. |
| O3138 | GAGAGGGGTACGAACTTGGTAGCGTCTCA |  |
| S3630 | CTTCTACTCTTGACACTGAATACTAC | To prepare DSB probe for centromere-proximal DSB detection |
| O3970 | TCTACTGGAACCATATTCACGGA |  |
